# A class of diffusive delayed viral infection models with general incidence function and cellular proliferation

**DOI:** 10.1007/s40065-022-00412-x

**Published:** 2022-12-05

**Authors:** Alexis Nangue, Willy Armel Tacteu Fokam

**Affiliations:** 1 Department of Mathematics, Higher Teachers’ Training College, University of Maroua, P.O.Box : 55, Maroua, Cameroon; 2 Department of Mathematics and Computer Sciences, Faculty of Sciences, University of Maroua, P.O.Box : 814, Maroua, Cameroon

**Keywords:** 34D23, 92B25, 37N25, 35Axx, 35Kxx

## Abstract

We propose and analyze a new class of three dimensional space models that describes infectious diseases caused by viruses such as hepatitis B virus (HBV) and hepatitis C virus (HCV). This work constructs a Reaction–Diffusion-Ordinary Differential Equation model of virus dynamics, including absorption effect, cell proliferation, time delay, and a generalized incidence rate function. By constructing suitable Lyapunov functionals, we show that the model has threshold dynamics: if the basic reproduction number $\mathcal {R}_{0}(\tau ) \le 1$, then the uninfected equilibrium is globally asymptotically stable, whereas if $\mathcal {R}_{0}(\tau ) > 1$, and under certain conditions, the infected equilibrium is globally asymptotically stable. This precedes a careful study of local asymptotic stability. We pay particular attention to prove boundedness, positivity, existence and uniqueness of the solution to the obtained initial and boundary value problem. Finally, we perform some numerical simulations to illustrate the theoretical results obtained in one-dimensional space. Our results improve and generalize some known results in the framework of virus dynamics.

## Introduction

Today, viral infection is linked to global health problems. Many diseases caused by viruses, such as hepatitis B virus (HBV), human immunodeficiency virus (HIV), hepatitis C virus (HCV), dengue virus, zika virus, and Sars-Cov-2 virus have drawn the attention of researchers to the viral infection process within a host. The mathematical modeling in a patient allows a better understanding of the transmission of diseases and thus improves the strategies for their eradication. On the basis of the virus infection models proposed in [1–3, 6, 9–13, 17–22, 24–35, 35–37], several mathematical models were examined and that are valuable for obtaining comprehensive knowledge about virus dynamics, for example models in the form of Ordinary Differential Equations (ODEs) [19–21, 34, 37], Delayed Differential Equations (DDE) [1, 22], Partial Differential Equations (PDE) [4, 29, 32, 35] and Fractional Differential Equations (FODE) [2, 3, 17, 25].

The basic reaction-diffusion-ODE viral infection dynamics model consists of the following three-dimensional system (see [4, 32] and references therein): 
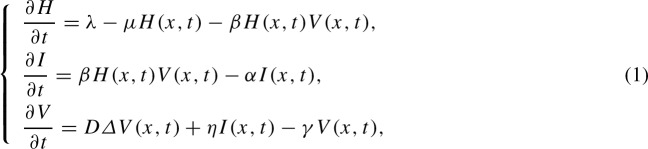
 where the density of uninfected cells is represented by *H*(*x*, *t*) at position *x* at time *t*, the density of infected cells is represented by *I*(*x*, *t*) at position *x* at time *t* and the density of free virus particles by *V*(*x*, *t*) at position *x* at time *t*. The uninfected target cells are produced at a constant rate $\lambda$ and are infected by free virus particles at a rate $\beta H(x,t)V(x,t)$ which follows mass action principle. The parameters $\mu$, $\alpha$ and $\gamma$ represent the death rates of uninfected cells, infected host cells and free virus particles, respectively. Free virions are produced by infected cells at the rate $\eta I(x,t)$. $\varDelta$ is the Laplacian operator and *D* is the diffusion coefficient.

At this point it should be mentioned that in model (1) it is assumed that the infection rate is bilinear, that is of the form $\beta H(x,t)V(x,t)$. However, this hypothesis does not always have a biological meaning. Recently, many researchers have performed virus dynamics models using various type of infection rate (or incidence function) which each time generalizes the bilinear infection rate. For example, in [35], authors studied a delayed model, in the case of HBV with diffusion and Holling-II infection rate, a virus infection model with the Crowley-Martin infection function has been studied in [16, 34, 37]. The Beddington-DeAngelis infection rate has been used in [10, 26, 36] to study a delayed in-host model with diffusion. Also in [27], authors studied a PDE-model with standard infection rate. Therefore, it is necessary to study the virus infection models with a more generalized infection rate, which can be represented by a function which has some properties and generalizes the later infection rates mentioned above.

In this work, motivated by the work done in [4], we further neglect the mobility of susceptible cells, infected cells, and we consider a delayed virus infection model with a generalized infection rate given as follows: 
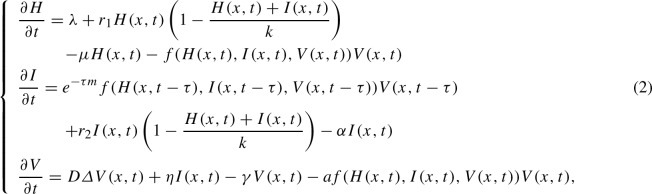
 for $t >0$ and $x \in \varOmega$ which is a bounded domain of $\mathbb {R}^{n}$ representing the liver with smooth boundary $\partial \varOmega$.

The original part of the model lies in the fact that the proliferation of cells due to mitotic division, and mitotic transmission obey a logistic growth. Thus the novelty of this model is that it includes both the intercellular delay in virus production, the proliferation of cells and the general incidence rate which generalizes most famous forms presented for instance in [14, 16, 26, 27, 34, 36, 37].

In model (2), we assume that the proliferation of cells due to mitotic division obeys a logistic growth law. The mitotic proliferation of uninfected cells is described by $$r_{1}H(x,t)\left( 1-\frac{H(x,t)+I(x,t)}{k}\right) $$, and mitotic transmission occurs at a rate $$r_{2}I(x,t)\left( 1-\frac{H(x,t)+I(x,t)}{k}\right) $$, which represents the mitotic division of infected cells. Some models supposed that infected hepatocytes do not proliferate; however, the effect of viral infection on hepatocytes is controversial, with conflicting data showing both proliferation induction and inhibition. With system of differential equations coupled to a reaction-diffusion equations models, we explore the impact of proliferation among infected cells in the liver. Uninfected cells and infected cells grow at the constant rate $$r_{1}$$ and $$r_{2}$$ respectively, and *k* is the maximal number of total cell population proliferation. The parameter $$ a \in \left\{ 0, 1 \right\} $$ indicates if there is an absorption effect or not. The infection process in its general form is characterized by the term *f*(*H*, *I*, *V*)*V*. In this case, the incidence function $$f = f(H, I, V) $$ is assumed to be continuously differentiable in the interior of $$\mathbb {R}^3_+$$ and probes the three assumptions given by Hattaf et al. [9, 15] and used in [11, 13, 31], that are:

$$(\mathcal {H}_{1})$$
: $$f(0,I,V)=0$$ for all $$I,V\ge 0$$;
$$(\mathcal {H}_{2})$$
: $$\frac{\partial f}{\partial H}(H,I,V)\ge 0$$ for all $$H,I,V\ge 0$$;
$$(\mathcal {H}_{3})$$
:$\frac{\partial f}{\partial I}(H,I,V)\le 0$ and $\frac{\partial f}{\partial V}(H,I,V)\le 0$ for all $I,V\ge 0$.

In the mathematical model (2) the immune response to infection is represented by an elevated death rate in infected cells, $\mu \le \alpha$, and by the destruction of free virions at rate $\gamma$. Due to the burden of supporting virus replication, infected cells may proliferate more slowly than uninfected cells, this implies that $r_{1} \le r_{2}$. The term $e^{-\tau m}f(H(x,t-\tau ), I(x,t-\tau ),V(x,t-\tau ))V(x,t-\tau )$ describes the newly activated infected cells at time *t* which are infected $\tau$ times ago. The recruitment of virus producing cells at time *t* is given by the number of cells that were newly infected at time $t - \tau$ and are still alive at time *t*. Here, *m* is assumed to be a constant death rate for infected but not yet virus-producing cells. Thus, the probability of surviving the time period from $t - \tau$ to *t* is $e^{-\tau m}$. We need a biologically reasonable history of the host for the system model. It is why the model (2) is supplemented with the following non-negative initial conditions:

3 $$\begin{aligned}{} & {} H(x,\theta )=\phi _{1}(x,\theta ) \ge 0, \; I(x,\theta )=\phi _{2}(x,\theta ) \ge 0, \; \nonumber \\{} & {} V(x,\theta )=\phi _{3}(x,\theta ) \ge 0, \; \theta \in [-\tau , 0],\; x\in \overline{\varOmega }, \end{aligned}$$

and homogeneous Neumann boundary condition

4 $$\begin{aligned} \frac{\partial V}{\partial n}=0 \; \text{ on }\; \partial \varOmega \times (0,+\infty ). \end{aligned}$$

It should be noted that the boundary condition in (4) imply that the free HCV virions do not move across the boundary $\partial \varOmega$.

In this paper we investigate the dynamical properties of the new model giving by (2), specifically the stability of the homogeneous equilibria. Our work is structured as follows. In the following section we discuss the existence and uniqueness, positivity and the boundedness of the solution to the IBVP with respect to the model. In Sect. 3, we start with the determination of the uninfected equilibrium, then followed by the determination of basic reproduction number $\mathcal {R}_{0}(\tau )$ and end with the study of the local and global asymptotic stability of this equilibrium. We first determine the infected equilibrium point and then we study the local and global asymptotic stability of this point in Sect. 4. The two previous sections are followed each of them by numerical simulations where we illustrate dynamic behaviour in more detail which reinforce the theoretical results. In the last section we give brief conclusion and perspectives.

## Preliminary results

This section is devoted to the study of existence, uniqueness, positivity and boundedness of solutions of the initial and boundary value problem (IBVP) (2)–(4). For this purpose, we first introduce the following spaces and definition: let $\mathbb {X}=\mathcal {C}(\overline{\varOmega }, \mathbb {R}^{3})$ be a Banach space of continuous functions from $\overline{\varOmega }$ to $\mathbb {R}^{3}$ and $\mathcal {C} = \mathcal {C}([-\tau , 0], \mathbb {X})$ be the Banach space of continuous functions from $[-\tau , 0]$ to $\mathbb {X}$ with the usual supremum norm and let $\mathcal {C}_{+} = \mathcal {C}([-\tau , 0], \mathbb {X}_{+})$ with $\mathbb {X}_{+}=\mathcal {C}(\overline{\varOmega }, \mathbb {R}^{3}_{+})$. We will say that $\varPhi \in \mathcal {C}$ if $\varPhi$ is a function from $\overline{\varOmega } \times [-\tau , 0]$ to $\mathbb {R}^{3}$ and is defined by $\varPhi (x, s) = \varPhi (s)(x)$. Also, we adopt the standard notation that for $\tau _{0} > 0$, a function $\varphi$: $[-\tau , \tau _{0}) \longrightarrow \mathbb {X}$ induces functions $\varphi _{t} \in \mathcal {C}$ for each $t \in [0, \tau _{0})$, defined by $\varphi _{t}(s) = \varphi (t + s)$, $s \in [-\tau ,0]$.

### Existence of local solution in time and positivity

For any data $\phi =(\phi _1,\phi _2,\phi _3)\in \mathcal {C}^{3}_+$, we define $F_i: \mathcal {C}_{+} \longrightarrow \mathbb {X}$, $i = 1, 2, 3$ as follows: for any $x\in \overline{\varOmega }$,

$$\begin{aligned} F_1(\phi )(x)= & {} \lambda +r_{1}\phi _1(x,0)\Bigg (1-\dfrac{\phi _{1}(x,0)+\phi _2(x,0)}{k}\Bigg )- \mu \phi _1(x,0)\\{} & {} -f(\phi _1(x,0),\phi _2(x,0),\phi _3(x,0))\phi _3(x,0),\\ F_2(\phi )(x)= & {} e^{-\tau m}f(\phi _1(x,-\tau ),\phi _2(x,-\tau ),\phi _3(x,-\tau ))\phi _3(x,-\tau )\\{} & {} +r_{2}\phi _2(x,0)\Bigg ( 1-\frac{\phi _1(x,0)+\phi _2(x,0)}{k}\Bigg ) -\alpha \phi _2(x,0),\\ F_3(\phi )(x)= & {} \eta \phi _2(x,0)-\gamma \phi _3(x,0)-f(\phi _1(x,0),\phi _2(x,0),\phi _3(x,0))\phi _3(x,0). \end{aligned}$$

Let $F=(F_1, F_2, F_3)$. We first prove that *F* is locally Lipschitz in $\mathbb {X}$.

#### Lemma 2.1

*F* is Lipschitz continuous on bounded subsets of $\mathcal {C}_{+}$.

#### Proof

Let $\varphi =(\varphi _1,\varphi _2,\varphi _3)\in \mathcal {C}^3_+$. Using the definition of $F_1$, we have:

5 $$\begin{aligned}{} & {} F_{1}\left( \phi _1,\phi _2,\phi _3\right) (x) -F_{1}\left( \varphi _1,\varphi _2,\varphi _3\right) (x) \nonumber \\{} & {} \quad = \frac{1}{2}\bigg (\left( f(\varphi _1(x,0),\varphi _2(x,0),\varphi _3(x,0)) -f(\phi _1(x,0),\phi _2(x,0),\phi _3(x,0))\right) (\varphi _3(x,0)+\phi _3(x,0))\nonumber \\{} & {} \qquad +(\varphi _3(x,0)-\phi _3(x,0))f(\varphi _1(x,0),\varphi _2(x,0),\varphi _3(x,0)) +f(\phi _1(x,0),\phi _2(x,0),\phi _3(x,0)) \bigg )\nonumber \\{} & {} \qquad + (r_1-\mu )(\phi _1(x,0)-\varphi _1(x,0)) +\dfrac{r_1}{k}\bigg ((\varphi _1(x,0)-\phi _1(x,0))(\phi _1(x,0)+\varphi _1(x,0))\bigg )\nonumber \\{} & {} \qquad + \dfrac{r_1}{k}\bigg (\varphi _1(x,0)\varphi _2(x,0)-\varphi _1(x,0)\phi _2(x,0)+\varphi _1(x,0)\phi _2(x,0) -\phi _1(x,0)\phi _2(x,0)\bigg ). \end{aligned}$$

From equality (5), it follows that

$$\begin{aligned}{} & {} \Vert F_{1}\left( \phi _1,\phi _2,\phi _3\right) (x) -F_{1}\left( \varphi _1,\varphi _2,\varphi _3\right) (x) \Vert _{\mathbb {X}} \\{} & {} \quad = \sup \limits _{x \in \overline{\varOmega }} \bigg | \frac{1}{2}\bigg (\left( f(\varphi _1(x,0),\varphi _2(x,0),\varphi _3(x,0)) -f(\phi _1(x,0),\phi _2(x,0),\phi _3(x,0))\right) (\varphi _3(x,0)+\phi _3(x,0))\\{} & {} \qquad +(\varphi _3(x,0)-\phi _3(x,0))f(\varphi _1(x,0),\varphi _2(x,0),\varphi _3(x,0)) +f(\phi _1(x,0),\phi _2(x,0),\phi _3(x,0)) \bigg )\\{} & {} \qquad + (r_1-\mu )(\phi _1(x,0)-\varphi _1(x,0)) +\dfrac{r_1}{k}\bigg ((\varphi _1(x,0)-\phi _1(x,0))(\phi _1(x,0)+\varphi _1(x,0))\bigg )\\{} & {} \qquad + \dfrac{r_1}{k}\bigg (\varphi _1(x,0)\varphi _2(x,0)-\varphi _1(x,0)\phi _2(x,0)+\varphi _1(x,0)\phi _2(x,0) -\phi _1(x,0)\phi _2(x,0)\bigg )\bigg |. \end{aligned}$$

According to the mean value theorem and assumptions made on *f*, there exists $M > 0$ and $\beta > 0$ such that employing (5) yields

$$\begin{aligned} \Vert F_{1}\left( \phi _1,\phi _2,\phi _3\right) (x) -F_{1}\left( \varphi _1,\varphi _2,\varphi _3\right) (x) \Vert _{\mathbb {X}}\le & {} \left( r_1 + MZ+ \frac{3Pr_1}{k}\right) \Vert \phi _1-\varphi _1\Vert _{\mathcal {C}}\\{} & {} +\left( \frac{P r_1}{k}+MZ\right) \Vert \phi _2-\varphi _2\Vert _{\mathcal {C}}\\{} & {} +(MZ+\beta )\Vert \phi _3-\varphi _3\Vert _{\mathcal {C}}, \end{aligned}$$

with *P* and *Z* given in theorem 2.4. In a similar manner, we get

$$\begin{aligned}{} & {} \Vert F_{2}\left( \phi _1,\phi _2,\phi _3\right) (x) -F_{2}\left( \varphi _1,\varphi _2,\varphi _3\right) (x) \Vert _{\mathbb {X}}\\{} & {} \quad \le \left( P\frac{r_2}{k}+MZe^{-m\tau }\right) \Vert \phi _1-\varphi _1\Vert _{\mathcal {C}} +\bigg (r_2+3\frac{r_2}{k}P +MZe^{-m\tau }\bigg )\Vert \phi _2-\varphi _2\Vert _{\mathcal {C}}\\{} & {} \qquad +(MZ+\beta )e^{-m\tau }\Vert \phi _3-\varphi _3\Vert _{\mathcal {C}}, \end{aligned}$$

and

$$\begin{aligned} \Vert F_{3}\left( \phi _1,\phi _2,\phi _3\right) (x) -F_{3}\left( \varphi _1,\varphi _2,\varphi _3\right) (x) \Vert _{\mathbb {X}}{} & {} \le aMP\Vert \phi _1-\varphi _1\Vert _{\mathcal {C}}+ (\eta +aMZ)\Vert \phi _2-\varphi _2\Vert _{\mathcal {C}}\\{} & {} +(\gamma +a(MZ+\beta ))\Vert \phi _3-\varphi _3\Vert _{\mathcal {C}}. \end{aligned}$$

Thus, *F* is Lipschitz continuous on bounded subsets of $\mathcal {C}_{+}$. $\square$

Now, IBVP (2)–(4) can be rewritten as the following abstract functional differential equation:

6 $$\begin{aligned} {\left\{ \begin{array}{ll} \dfrac{d\omega }{\textrm{d}t} = A\omega +F(\omega _{t}), \;\; t>0, \;\; \omega _{t} \in \mathcal {C}, \\ \omega (0)=\phi \in \mathcal {C}_+, \end{array}\right. } \end{aligned}$$

where $$\omega =(H,I,V)$$, $$ \phi =(\phi _1,\phi _2,\phi _3)$$ and $$A\omega =(0,0,D\varDelta V)^{\top }$$.

Let $\mathbb {O}=(0,0,0)^{\top }$ and $N=(T_1, T_2, T_3)^{\top }$ where $T_1=\frac{(r_1-\mu )+\sqrt{(r_1-\mu )^{2}+4\frac{r_1}{k}\lambda }}{\frac{2r_1}{k}}$, $T_2=\frac{(r_2-\alpha )+\sqrt{(r_2-\alpha )^{2}+4e^{-m\tau }T_1\beta _2\frac{r_2}{k}}}{\frac{2r_2}{k}}$, $T_3=\frac{T_2\eta }{\gamma }$ with $J_1=\sqrt{(r_1-\mu )^{2}+4\lambda \frac{r_1}{k}}$, $J_2= \sqrt{(r_2-\alpha )^{2}+4e^{-m\tau }\beta _{2}T_{1}\frac{r_2}{k}}$. Let us also consider the following sets

$$\begin{aligned}{} & {} {[}\mathbb {O},\,N{]}_\mathbb {X}=\{\phi \in \mathbb {X}^{3}_+: \, 0\le \phi (x)\le N : \forall \,x\in \overline{\varOmega } \}; \\{} & {} {[}\mathbb {O},\,N{]}_\mathcal {C}=\{\phi \in \mathcal {C}^{3}_+: \, \phi (\theta )\in [\mathbb {O},N]_\mathbb {X} : \forall \,\theta \in [-\tau , 0] \}. \end{aligned}$$

We prove the following result:

#### Lemma 2.2

For any $$\phi \in [\mathbb {O},\,N]_\mathcal {C}$$, $$\lim \limits _{\rho \rightarrow +0}\dfrac{1}{\rho }dist\bigg ( \phi (0)+\rho F(\phi ), \, [\mathbb {O}, \,N]_\mathbb {X}\bigg ) =0$$.

#### Proof

For $\phi \in [\mathbb {O},\,N]_\mathcal {C}$ and for any $0\le \rho \le \min \bigg \{\frac{1}{\mu +\beta },\, \frac{1}{\alpha },\,\frac{1}{\gamma +aT_1\beta },\, \frac{2}{(r_1+\mu )+J_1},\, \frac{2}{(r_2+\alpha )+J_2 } \bigg \}$, we have

$$\begin{aligned} \phi (x,0)+\rho F(\phi )(x)\ge & {} \left( \begin{array}{c} \phi _1(x,0)-\rho \mu \phi _1(x,0)-\rho \beta \phi _1(x,0) \\ \phi _2(x,0)-\rho \alpha \phi _2(x,0)\\ \phi _3(x,0)-\rho \gamma \phi _3(x,0)-\rho a\beta T_1\phi _3(x,0)\\ \end{array} \right) \\\ge & {} \left( \begin{array}{c} (1-\rho (\mu +\beta _2))\phi _1(x,0) \\ (1-\rho \alpha )\phi _2(x,0)\\ (1-\rho (\gamma +aT_1\beta )\phi _3(x,0) \end{array} \right) \ge \left( \begin{array}{c} 0\\ 0\\ 0 \end{array} \right) =\mathbb {O}. \end{aligned}$$

Moreover, we can easily obtain:

$$\begin{aligned}&\phi (x,0)+\rho F(\phi )(x)\\&\quad \le \left( \begin{array}{c} \phi _1(x,0)+\rho \lambda +\rho r_{1}\phi _1(x,0)\bigg (1-\dfrac{\phi _1(x,0)}{k}\bigg )-\rho \mu \phi _1(x,0) \\ \phi _2(x,0)+\rho e^{-m\tau }\beta _1\phi _1(x,0)+\rho r_{2}\phi _2(x,0)\bigg (1-\dfrac{\phi _2(x,0)}{k}\bigg )-\rho \alpha \phi _2(x,0)\\ \phi _3(x,0)+\rho \eta \phi _2(x,0)-\rho \gamma \phi _3(x,0) \end{array} \right) \\&\quad \le \left( \begin{array}{c} T_1\\ T_2\\ T_3 \end{array} \right) = N. \end{aligned}$$

We have now shown that for $\rho$ small enough,

$$\begin{aligned}\phi (0)+\rho F(\phi )\in [\mathbb {O},\,N]_\mathbb {X}, \end{aligned}$$

from which we deduce that

$$\begin{aligned} \lim _{\rho \rightarrow 0^{+}}\frac{1}{\rho }dist(\phi (0)+\rho F(\phi ), \, [\mathbb {O},\,N]_\mathbb {X})=0, \,\, \, \forall \phi \in [\mathbb {O}\,,N]_\mathcal {C}. \end{aligned}$$

$$\square $$

From the main results of the literature and the previous lemmas, we can state this following result.

#### Theorem 2.3

For any $\phi = (\phi _{1}, \phi _{2}, \phi _{3})\in [\mathbb {O},\,N]_\mathcal {C}$, there exists a unique nonnegative solution $\varphi (t, x; \phi )$ of the IBVP (4)–(2) defined for $t \in [0, +\infty )$. Furthermore $\varphi _{t} \in [\mathbb {O}, \,N ]_\mathcal {C}$ for $t \ge 0$.

#### Proof

For $\mathbb {D}=(0,0,D)^{\top }$, according to Theorem 1.5 of [5] the $\mathbb {X}$-realisation of the operator $\mathbb {D}\varDelta$ generates an analytical semi-group *T*(*t*) on $\mathbb {X}$. Applying the Corollary 4 of [18], we conclude that the IVBP (2)–(4) admits a unique mild solution $\varphi (t,\,\phi )=(H(t,\,\phi ),I(t,\,\phi ),v(t,\,\phi ))\in [\mathbb {O},\ N]_{\mathcal {C}}$ for $t\in [0,\,+\infty )$. In addition, Corollary 2.5 of [33] ensures that the mild solution is classic for $t\ge \tau .$ $\square$

### Boundedness of the solutions of the IBVP (2)–(4)

In this section, we establish the boundedness in time of the global solution of the IBVP (2)–(4) for $$ x \in \overline{\varOmega }$$ and $$t \in [0, T_{max} ) $$ where $$T_{max} > 0 $$ is the maximal existence time for solution of the the IBVP (2)–(4).

#### Theorem 2.4

For any solution (*H*, *I*, *V*) of problem (2)–(4),

$$\begin{aligned} H(x,t)\le P, \;\; I(x,t)\le P \;\; \text{ and } \;\; V(x,t)\le Z \end{aligned}$$

for all $(x,t) \in \overline{\varOmega } \times [-\tau , T_{max} )$ where

$$\begin{aligned} P=\max \left\{ \frac{4\lambda +(r_1+r_2)k}{4b};\max _{x\in \overline{\varOmega }}\left\{ \phi _1(x,0)+\phi _2(x,0)+\int _{-\tau }^{0}e^{ms}f(H(x,s),I(x,s),V(x,s))V(x,s)ds\right\} \right\} \end{aligned}$$

and

$$\begin{aligned} Z=\max \bigg \{\frac{\eta P}{\gamma }; \, \max _{x\in \overline{\varOmega }}^{}\phi _3(x,0) \bigg \} \end{aligned}$$

with

$$\begin{aligned} b=\min \left\{ \mu , \alpha , m \right\} . \end{aligned}$$

#### Proof

Let (*H*, *I*, *V*) be a solution of problem (2)-(4). Let us define the following function

$$\begin{aligned} U(x,t)=H(x,t)+I(x,t)+\int _{t-\tau }^{t}e^{-m(t-s)}f(H(x,s),I(x,s),V(x,s))V(x,s)ds, \end{aligned}$$

for $x \in \overline{\varOmega }$ and $t \in [0, T_{max} )$. We obtain, after some lengthy calculations,

$$\begin{aligned} \frac{\partial U(x,t)}{\partial t}= & {} \lambda + \left( \left( H(x,t)+I(x,t)\right) \left( r_1+r_2\right) \right) \left( 1 - \dfrac{H(x,t) + I(x,t)}{k}\right) \\{} & {} - \left( r_{1}I(x,t)+r_2 H(x,t)\right) \left( 1 - \dfrac{H(x,t) + I(x,t)}{k}\right) - \mu H(t)\\{} & {} -m \int _{t-\tau }^{t} e^{-m(t-s)}f(H(x,s),I(x,s),V(x,s))V(x,s) ds-\alpha I(x,t)\\= & {} \lambda +\left( \frac{r_1+r_2}{4}\right) k- \left( \frac{r_1+r_2}{k}\right) \left[ H(x,t)+I(x,t) - \frac{k}{2}\right] ^{2} \\{} & {} - \left( r_{1}I(t)+r_2 H(x,t)\right) \left( 1 - \dfrac{H(x,t) + I(x,t)}{k}\right) - \mu H(x,t)\\{} & {} -m \int _{t-\tau }^{t} e^{-m(t-s)}f(H(x,s),I(x,s),V(x,s))V(x,s) ds-\alpha I(x,t). \end{aligned}$$

Due to

$$\begin{aligned} \left( \frac{r_1+r_2}{k}\right) \left[ H(x,t)+I(x,t) - \frac{k}{2}\right] ^{2}\ge 0, \end{aligned}$$

and

$$\begin{aligned} \left( r_{1}I(x,t)+r_2 H(x,t)\right) \left( 1 - \dfrac{H(x,t) + I(x,t)}{k}\right) \ge 0, \end{aligned}$$

it follows that

$$\begin{aligned}{} & {} \frac{\partial U(x,t)}{\partial t}\le \lambda +\left( \frac{r_1+r_2}{4}\right) k- \mu H(x,t)-\alpha I(x,t) -m\beta \\{} & {} \quad \int _{t-\tau }^{t} e^{-m(t-s)} f(H(x,s),I(x,s),V(x,s))V(x,s)ds. \end{aligned}$$

Hence

7 $$\begin{aligned} \frac{\partial U(x,t)}{\partial t} \le \lambda + \left( \frac{r_1+r_2}{4}\right) k-bU(x,t). \end{aligned}$$

with

$$\begin{aligned} b=\min \left\{ \mu , \alpha , m \right\} . \end{aligned}$$

From (7), we get:

8 $$\begin{aligned} U(x,t)\le \max \bigg \{\frac{4\lambda +(r_1+r_2)k}{4b},\,\max _{x\in \overline{\varOmega }}U(x,0)\bigg \}. \end{aligned}$$

Now to have the bounds of *V*, we consider the following problems

$$\begin{aligned} {\left\{ \begin{array}{ll} \dfrac{\partial V(x,t)}{\partial t}- D\varDelta V(x,t) = \eta I(x,t) - \gamma V(x,t)-af(H,I,V)V, \; \text{ on } \;\varOmega \times (0,\,\,+\infty ), \\ \le D\varDelta V(x,t)+\eta P-\gamma V(x,t), \; \text{ on }\;\varOmega \times (0,\,\,+\infty ),\\ \frac{\partial V(x,t)}{\partial n}=0, \; \text{ on } \; \partial \varOmega \times (0,\,\,+\infty ),\\ V(x,0)=\phi _3(x,0), \; x\in \varOmega , \end{array}\right. } \end{aligned}$$

and

$$\begin{aligned} {\left\{ \begin{array}{ll} \dfrac{\partial \overline{V}(t)}{\partial t}= \eta P-\gamma \overline{V}(t) \,\,\, \,\text{ on } \,\,\, \, (0,\,\,+\infty ), \\ \overline{V}(0)=\max \limits _{x\in \overline{\varOmega }}^{}\phi _3(x,0). \end{array}\right. } \end{aligned}$$

Using a comparison principle (see [23]), we infer that

$$\begin{aligned} V(x,t)\le \overline{V}(t), \end{aligned}$$

where

$$\begin{aligned} \overline{V}(t)\le \max \bigg \{\frac{\eta P}{\gamma }; \, \max _{x\in \overline{\varOmega }}^{}\phi _3(x,0) \bigg \}. \end{aligned}$$

This implies that *V* is bounded on $\overline{\varOmega }\times [0,\,T_{\max })$.

From the above discussion, we deduce that *H*, *I* and *V* are bounded on $\overline{\varOmega }\times [0,\,T_{\max })$. $\square$

## Asymptotic stability analysis of the uninfected equilibrium

The aim of this section is to study the local and global stability of the uninfected equilibrium.

### Basic reproduction number and Uninfected equilibrium

It is easy to verify that system (2) always has an uninfected equilibrium $$E_{0} =(H_{0}, 0, 0) $$ with

$$\begin{aligned} H_0=\left[ \left( r_1-\mu \right) +\left( (r_1-\mu )^2+4\frac{r_1 \lambda }{k}\right) ^{1/2}\right] \dfrac{k}{2r_1}. \end{aligned}$$

Following Wang and Zhao [30], we define the basic reproduction number of our model in the absence of spatial dependence as follows:

$$\begin{aligned} \mathcal {R}_{0}(\tau )=\frac{1}{\alpha }\bigg [ r_{2}\bigg (1-\frac{H_{0}}{k}\bigg )+\frac{\eta e^{-\tau m}f(H_{0},0,0)}{\gamma + af(H_0,0,0)}\bigg ]. \end{aligned}$$

One of the main tools in epidemic models is the basic reproduction number which is an important threshold parameter to discuss the dynamic behaviour of the epidemic model. It quantifies the infection risk. It measures the expected average number of new infected hepatocytes generated by a single virion in a completely healthy hepatocyte.

### Local stability analysis of $E_{0}$

The objective of this section is to prove the local stability of the spatially homogeneous uninfected equilibrium $E_{0}$ for the Reaction-Diffusion-ODE system (2). We address local stability by analyzing the characteristic equation.

#### Proposition 3.1

The spatially homogeneous uninfected equilibrium $E_{0}$ of Reaction-Diffusion-O.D.E model system (2) is locally asymptotically stable if $\mathcal {R}_{0}(\tau ) < 1$ and it is unstable if $\mathcal {R}_{0}(\tau ) > 1$.

#### Proof

Let $\{\mu _{l}, \varphi _{l}\}$ be an eigenpair of the Laplace operator $-\varDelta$ on $\varOmega$ with the homogeneous Neumann boundary condition where $0=\mu _{1}<\mu _{2}<\mu _{3}< \cdot \cdot \cdot$ $\rightarrow$ $\infty$. Let $E_{\mu _{l}}$ be the eigenspace corresponding to $\mu _{l}$ in $C^{1}(\varOmega )$ and $\{ \varPhi _{lj}, j=1,2,\cdot \cdot \cdot ,\text{ dim } E_{\mu _{l}} \}$ be an orthogonal basis of $E_{\mu _{l}}$. Let $\mathbb {Y}=(C^{1}(\varOmega ))^{3}$ and $\mathbb {X}_{lj}=\{ c \varphi _{lj},\;/ \; c\in \mathbb {R}^{3} \}$.

Consider the following direct sum

$$\begin{aligned} \mathbb {Y}=\bigoplus \limits _{l=1}^{\infty }\mathbb {X}_{l} \;\; \text{ with }\;\; \mathbb {X}_{l}=\bigoplus \limits _{j=1}^{dim E_{\mu _{l}}}\mathbb {X}_{lj}, \end{aligned}$$

where $\mathbb {X}_{lj}$ is the eigenspace corresponding to $\mu _{l}$. The linearization of system (2) at the spatially homogeneous uninfected equilibrium $E_{0}$ can be formulated by:

9 $$\begin{aligned} \frac{\partial z}{\partial t} = \overline{D} \varDelta z + \mathcal {A} z + \mathcal {B}z_{t}, \end{aligned}$$

where $$ z = (H, I, V ) $$, $$ z_{\tau } = (H_{\tau }, I_{\tau }, V_{\tau } ) $$, $$\overline{D}=diag(0,0,D)$$,

$$\mathcal {A}=\left( \begin{array}{ccc} r_{1}\left( 1 - \frac{2H_0}{k}\right) - \mu &{}-\dfrac{r_1}{k}H_0&{}-f(H_0,0,0)\\ 0&{}r_{2}\left( 1 - \frac{H_0}{k}\right) -\alpha &{}0\\ 0&{}\eta &{}-\gamma -af(H_0,0,0) \end{array}\right) $$

and

$$\mathcal {B}=\left( \begin{array}{ccc} 0&{}0&{}0\\ 0&{}0&{} e^{-\tau m}f(H_0,0,0) \\ 0&{}0&{}0 \\ \end{array}\right) .$$

For each *i* = 0, 1, 2 $\cdot \cdot \cdot$, $\mathbb {X}_{l}$ is invariant under the linearization. We use the exponential Ansatz $z(x, t) = e^{st} \varPhi (x)$ where $\varPhi \in \mathbb {X}_{l}$ satisfies $\varDelta \varPhi = - \mu _{i} \varPhi$. Then, we find that $z_{\tau }=z(x, t-\tau ) = e^{-X \tau } z(x,t)$. Noting that $\frac{\partial z}{\partial t} = X z$, we substitute into (9) obtaining

$$\begin{aligned} Xz=-\mu _{i}\overline{D} z+\mathcal {A} z+e^{-\tau X} \mathcal {B} z. \end{aligned}$$

Letting *Id* be the $3 \times 3$ identity matrix, we have

10 $$\begin{aligned} ( -XId -\mu _{i}\overline{D} +\mathcal {A} +e^{-\tau X} \mathcal {B}) z = 0. \end{aligned}$$

Thus, there exist non-trivial solutions *z* and *X* is an eigenvalue if the matrix $-XId -\mu _{i}\overline{D} +\mathcal {A} +e^{-\tau X} \mathcal {B}$ has a determinant equal to zero. This calculation gives the characteristic equation as follows:

11 $$\begin{aligned}{} & {} - \left( X - \left( r_1 - \mu -2 \frac{r_1}{k}H_0\right) \right) \left( X^2 + \left( \frac{r_{2}}{k}H_0 + \gamma +af(H_0,0,0) + \alpha - r_2 +\mu _{i}D \right) X \right) \nonumber \\{} & {} + \bigg ( -r_2 \gamma + \alpha \gamma + \frac{r_2}{k} H_0 \gamma -r_2af(H_0,0,0)+\alpha af(H_0,0,0)+ \frac{r_2}{k} H_0 af(H_0,0,0) \nonumber \\{} & {} -\mu _{i}D(r_2-\frac{r_2H_0}{k}-\alpha ) - \eta e^{(m+X)\tau }f(H_0,0,0) \bigg ) = 0. \end{aligned}$$

Considering that $E_{0}$ verifies system (17), hence $r_1 \left( 1 - \frac{H_0}{k} \right) = \mu - \frac{\lambda }{H_0}$, and using the previous fact we can express the first factor of (11) as:

$$\begin{aligned} X= r_1 -2 \frac{r_1}{k} H_0 - \mu =- \left( \frac{\lambda }{H_0} + \frac{r_1 H_0}{k}\right) , \end{aligned}$$

which have a negative eigenvalue, and the other two eigenvalues satisfy the following transcendental polynomial

12 $$\begin{aligned} X^2 + a_2X + a_3 + b_3 (\tau ) e^{-X\tau } = 0, \end{aligned}$$

where

$$\begin{aligned}{} & {} a_2 =\frac{r_2}{r_1}\frac{\lambda }{H_0} - \frac{r_2}{r_1} \mu + \gamma +af(H_0,0,0)+ \alpha +\mu _{i}D, \\{} & {} a_{3}=-r_2 \left( \gamma +af(H_0,0,0)\right) \left( 1 - \frac{H_0}{k}\right) + \alpha \gamma +\alpha af(H_0,0,0) -\mu _{i}D(r_2-\frac{r_2 H_0}{k}-\alpha ),\\{} & {} b_3 (\tau ) = - \eta e^{-m \tau } f(H_0,0,0). \end{aligned}$$

It is clear that $a_{2} > 0$ due to the fact that $r_2 \le r_1$ and $\mu \le \alpha$.

When $\tau =0$, equation (12) becomes

13 $$\begin{aligned} X^2 + a_2 X + a_3 + b_3(0)=0. \end{aligned}$$

We have

$$\begin{aligned} a_3 + b_3(0)= & {} -r_2\left( \gamma +af(H_0,0,0)\right) \left( 1 - \frac{H_0}{k}\right) + \alpha \gamma +\alpha af(H_0,0,0)\\{} & {} -\mu _{i}D\left( r_2-\frac{r_2 H_0}{k}-\alpha \right) - \eta f(H_0,0,0)\\= & {} -\alpha \left( \gamma +af(H_0,0,0) \right) \left[ \frac{r_2}{\alpha }\left( 1 - \frac{H_0}{k}\right) -\frac{\eta f(H_0,0,0)}{\alpha \left( \gamma +af(H_0,0,0)\right) }-1\right] \\{} & {} -\mu _{i}D(r_2-\frac{r_2 H_0}{k}-\alpha )\\= & {} -\alpha \left( \gamma +af(H_0,0,0)\right) \left[ \mathcal {R}_0 (0)-1\right] -\mu _{i}D\left[ -\frac{r_2}{r_1}\frac{\lambda }{H_0} + \frac{r_2}{r_1} \mu -\alpha \right] . \end{aligned}$$

If $\mathcal {R}_0 (0) < 1$ then $a_3 + b_3(0)>0.$ Furthermore, the fact that $a_3 >0$, ensures that all the roots of (13) have negative real part according to Routh-Hurwitz criterion. Therefore, the uninfected equilibrium $E_{0}$ is locally asymptotically stable when $\tau =0$.

Now, let us consider the distribution of the roots of (12) when $\tau > 0$.

Assume that $X=\omega i$ $(\omega >0 )$ is a solution of (12). Substituting $X=\omega i$ $(\omega >0 )$ into (12), then separating in real and imaginary parts, we obtain

$$\begin{aligned} \left\{ \begin{array}{ccc} a_3 - \omega ^2&{}=&{}-b_3 (\tau ) \cos \omega \tau ,\\ a_2 \omega &{}=&{} b_3 (\tau ) \sin \omega \tau . \end{array} \right. \end{aligned}$$

Squaring and adding the last two equations and after simplifications yield

$$\begin{aligned} \omega ^4 + (a_2^2 - 2a_3) \omega ^2 + (a_3^2 - b_3^2(\tau ))=0. \end{aligned}$$

Let

$$\begin{aligned} \omega ^2 = Z; \quad A = a_2^2 - 2 a_3;\quad B(\tau )=a_3^2 - b_3^2(\tau ), \end{aligned}$$

then, we have

14 $$\begin{aligned} F(Z) = Z^2 + AZ + B(\tau )=0, \end{aligned}$$

where

$$\begin{aligned} A= & {} \bigg (\frac{r_2 \lambda }{r_1 H_0} - \frac{r_2}{r_1}\mu + \alpha \bigg )^{2}+ \left( \gamma +af(H_0,0,0)\right) ^2+2\mu _{i}D\left( \gamma +af(H_0,0,0)\right) +(\mu _{i}D)^{2} > 0. \end{aligned}$$

and

$$\begin{aligned} B(\tau ) = a_3^2 - b_3^2(\tau ) = \left( a_3 - b_3(\tau )\right) \left( a_3 + b_3(\tau )\right) . \end{aligned}$$

where

$$\begin{aligned} \left( a_3 + b_3(\tau )\right) =-\alpha \gamma \left[ \mathcal {R}_{0}(\tau )-1\right] -\mu _{i}D\left[ \frac{r_2}{r_1}\frac{\lambda }{H_0} - \frac{r_2}{r_1} \mu -\alpha \right] . \end{aligned}$$

Additionally,

$$\begin{aligned} a_3 + b_3(\tau )= & {} -r_2\left( \gamma +af(H_0,0,0)\right) \left( 1 - \frac{H_0}{k}\right) + \alpha \gamma +\alpha af(H_0,0,0) \\{} & {} \quad -\mu _{i}D(r_2-\frac{r_2 H_0}{k}-\alpha )+\eta e^{-m \tau } f(H_0,0,0)\\= & {} \left( \gamma +af(H_0,0,0)\right) \bigg [\frac{r_2 \lambda }{r_1 H_0} - \frac{r_2}{r_1}\mu + \alpha \bigg ]-\mu _{i}D\left[ -\frac{r_2}{r_1}\frac{\lambda }{H_0} + \frac{r_2}{r_1} \mu -\alpha \right] \\{} & {} \quad +\eta e^{-m \tau } f(H_0,0,0). \end{aligned}$$

Therefore,

$$\begin{aligned} a_3 + b_3(\tau )= & {} \left( \gamma +af(H_0,0,0)\right) \bigg [\frac{r_2 \lambda }{r_1 H_0} - \frac{r_2}{r_1}\mu + \alpha \bigg ]-\mu _{i}D\left[ -\frac{r_2}{r_1}\frac{\lambda }{H_0} + \frac{r_2}{r_1} \mu -\alpha \right] \\{} & {} \quad +\eta e^{-m \tau } f(H_0,0,0). \end{aligned}$$

Since $\frac{r_2 \mu }{r_1} -\alpha <0$, if $\mathcal {R}_0(\tau )<1$, then $B(\tau )>0$.

Now as $A>0$, $B(\tau )>0$ and $\omega >0$, then $F(Z)>0$ for any $Z>0$, which contradicts $F(Z)=0.$ This shows that characteristic equation (14) does not have pure imaginary roots when $\mathcal {R}_0(\tau )<1$. The fact that $E_{0}$ is locally asymptotically stable for $\tau = 0$, and the continuity of the roots of (14) with respect to the delay imply that (14) has all its roots with real negative part when $\mathcal {R}_0(\tau )<1$.

Therefore, if $\mathcal {R}_0(\tau )<1$, the uninfected equilibrium $E_{0}$ of system (2) is locally asymptotically stable.

Now we consider the case $\mathcal {R}_0(\tau )>1$, recalling that *i* specifies the diffusion eigenvalue $\mu _{i}$, let

15 $$\begin{aligned} F(X, i) = X^2 + a_2X + a_3 + b_3 (\tau ) e^{-X\tau }. \end{aligned}$$

Here, it is sufficient to consider $$i = 0 $$ and the space $$\mathbb {X}_{0}$$ corresponding to $$ \mu _{0} = 0 $$. We have

$$\begin{aligned} F(0,0) = -\alpha \gamma \left[ \mathcal {R}_0(\tau )-1\right] < 0 \end{aligned}$$

and

$$\begin{aligned} \lim \limits _{X \rightarrow + \infty }F(X, 0) = + \infty \end{aligned}$$

Hence, there must exist $$ X_{0} > 0 $$ such that $$F(X_{0}, 0) =0 $$. This yields that (13) has at least one positive root. Thus, the uninfected equilibrium $$E_{0}$$ is unstable for $$\mathcal {R}_0(\tau )>1$$. $$\square $$

### Global stability analysis of $E_{0}$

We discuss about the global stability of the uninfected equilibrium $E_0 =(H_0,0,0)$ for the delayed Reaction-Diffusion-O.D.E model problem (2)–(4) when $\mathcal {R}_0(\tau )<1$. We set

$$\begin{aligned} y=y(x,t)\quad \text{ and }\quad y_{\tau }=y(x,t-\tau )\;\; \text{ forall } \;\; y\in \{H,I,V\}. \end{aligned}$$

We assume that *f* is under the following hypothesis:

$$\begin{aligned} (\mathcal {H}_{4}): \exists \delta> 0\;\; \text{ such } \text{ that } \;\; \left( 1-\frac{f(H_0,0,0)}{f(H,0,0)}\right) =\frac{\delta (H-H_0)}{H}, \;\;\text{ for } \text{ all }\;\; H>0. \end{aligned}$$

We have the following result:

#### Theorem 3.2

Suppose that $r_1 = r_{2}\frac{\left( \gamma +af(H_0,0,0)\right) }{\gamma \delta }e^{\tau m}$ and if $\mathcal {R}_0(\tau )\le 1$, then the uninfected equilibrium $E_0 =(H_0,0,0)$ of the delayed Reaction-Diffusion-O.D.E model problem (2)–(4) is globally asymptotically stable.

#### Proof

Let $H=H(x,t)$, $I=I(x,t)$ and $V=V(x,t)$ be any positive solution of the delayed Reaction-Diffusion-O.D.E model problem (2)–(4). Define the following Lyapunov functional

16 $$\begin{aligned} L_{1}(t)= & {} \int _{\varOmega } \left( r_2e^{m\tau }\left( H-H_0- \int _{H_0}^{H} \frac{f(H_0,0,0)}{f(\eta ,0,0)} \textrm{d}\eta \right) + e^{m\tau } r_ 1\delta I + \frac{r_1 \delta f (H_0,0,0) V}{\gamma +af(H_0,0,0) }\right) \textrm{d}x \nonumber \\{} & {} +r_1 \delta \int _{\varOmega }\int _{t-\tau }^{t} f(H(x,u),I(x,u),V(x,u))V(x,u)\textrm{d}u\textrm{d}x. \end{aligned}$$

Then, clearly, $L_{1}(t)$ is nonnegative definite with respect to $E_{0}$. Now, calculating the time derivative of $L_{1}$ along the solution of problem (2)–(4), where the dot $(^{\cdot })$ represents derivative with respect to time, we obtain

$$\begin{aligned} \frac{d L_{1}(t)}{\textrm{d}t}= & {} \int _{\varOmega } \left( r_2e^{m\tau }\left( 1-\frac{f(H_0,0,0)}{f(H,0,0)} \right) \dot{H} + e^{m\tau } r_ 1\delta \dot{I} + \frac{r_1 \delta f (H_0,0,0) V}{\gamma +af(H_0,0,0) }\dot{V}\right) \textrm{d}x \\{} & {} +r_1 \int _{\varOmega }\delta \frac{d}{\textrm{d}t}\int _{t-\tau }^{t} f(H(x,u),I(x,u),V(x,u))V(x,u)\textrm{d}u\textrm{d}x \\= & {} \int _{\varOmega }^{}\bigg [e^{m\tau } r_2 \frac{\delta (H-H_0)}{H}\left( \lambda + r_1 H\left( 1- \frac{H+I}{k}\right) -\mu H \right) \\{} & {} -e^{m\tau } r_2\left( 1-\frac{f(H_0,0,0)}{f(H,0,0)}\right) f(H,I,V)V +r_1 \delta f(H_{\tau },I_{\tau } V_{\tau })V_{\tau }\\{} & {} +r_1 e^{m\tau } r_2 \delta I\left( 1-\frac{H+I}{k}\right) -r_1 \delta \alpha e^{m\tau }I - r_1 \delta f(H_{\tau },I_{\tau },V_{\tau })V_{\tau } + r_1 \delta f(H,I,V)V\\{} & {} +\frac{r_1 \delta f(H_0,0,0) \eta I}{\gamma +af(H_0,0,0)} - \frac{r_1 \delta f(H_0,0,0)V\gamma }{\gamma +af(H_0,0,0)}-\frac{r_1 \delta af(H_0,0,0)f(H,I,V)V}{\gamma +af(H_0,0,0)}\\{} & {} +D\frac{r_1 \delta f(H_0,0,0)}{\gamma +af(H_0,0,0)}\varDelta V\bigg ]\textrm{d}x. \end{aligned}$$

Since

$$\begin{aligned} r_1 - \mu =\frac{r_1 H_0}{k}-\frac{\lambda }{H_0}, \end{aligned}$$

we have

$$\begin{aligned} \frac{d L_{1}(t)}{\textrm{d}t}= & {} \int _{\varOmega }^{}\bigg [e^{m\tau } r_2 \frac{\delta (H-H_0)}{H}\left( \lambda + \frac{r_1}{k} H_0 H -\frac{\lambda }{H_0}H - \frac{r_1}{k}IH - \frac{r_1}{k}H^{2}\right) \\{} & {} \quad -e^{m\tau } r_2\left( 1-\frac{f(H_0,0,0)}{f(H,0,0)}\right) f(H,I,V)V +e^{m\tau }Ir_1r_2\delta \left( 1-\frac{H_0}{k}\right) \\{} & {} \quad -e^{m\tau }Ir_1r_2\delta \left( \frac{H-H_0}{k}\right) -r_1e^{m\tau }r_2\frac{I^2}{k}\delta -r_1\delta \alpha e^{m\tau }I+\frac{r_1 \delta f(H_0,0,0) \eta I}{\gamma +af(H_0,0,0)}\\{} & {} \quad - \frac{r_1 \delta f(H_0,0,0)V\gamma }{\gamma +af(H_0,0,0)}+\frac{r_1 \delta f(H,I,V)V\gamma }{\gamma +af(H_0,0,0)}+D\frac{r_1 \delta f(H_0,0,0)}{\gamma +af(H_0,0,0)}\varDelta V\bigg ]\textrm{d}x\\= & {} \int _{\varOmega }^{}\bigg [ - \frac{r_1r_2\delta }{H_0}e^{m \tau }\left( (H-H_0)+I\right) ^2 + e^{m\tau } r_1 \delta \alpha I(\mathcal {R}_0(\tau )-1) \\{} & {} \quad -\lambda \delta e^{m \tau } \frac{r_2 (H-H_0)^2}{HH_0} -e^{m\tau } r_2f(H,I,V)V+e^{m\tau } r_2\frac{f(H_0,0,0)}{f(H,0,0)}f(H,I,V)V \\{} & {} \quad - \frac{r_1 \delta f(H_0,0,0)V\gamma }{\gamma +af(H_0,0,0)}+ \frac{r_1 \delta \gamma f(H,I,V)V}{\gamma +af(H_0,0,0)}+D\frac{r_1 \delta f(H_0,0,0)}{\gamma +af(H_0,0,0)} \varDelta V\bigg ]\textrm{d}x. \end{aligned}$$

Moreover, using the fact *f* is a decreasing function with respect to second and third variable,

$$\begin{aligned} f(H,I,V)\le f(H,0,0) \;\; \text{ forall }\;\; H,\,I,\,V\ge 0, \end{aligned}$$

hence it follows that

$$\begin{aligned} \frac{\partial L_{1}(t)}{\partial t}\le & {} \int _{\varOmega }^{}\bigg [-\lambda \delta e^{m\tau } r_2 \frac{(H-H_0)^2}{HH_0} -\frac{r_1 \delta }{k}r_2e^{m\tau }\left( (H-H_0) + I \right) ^2 \\{} & {} + e^{m\tau } r_1 \delta \alpha I (\mathcal {R}_0(\tau ) -1)-f(H,I,V)V\left( e^{m\tau } r_2-\frac{r_1 \delta \gamma }{\gamma +af(H_0,0,0)}\right) \\{} & {} +D\frac{r_1 \delta f(H_0,0,0)}{\gamma +af(H_0,0,0)} \varDelta V+f(H_0,0,0)V\left( e^{m\tau } r_2-\frac{r_1 \delta \gamma }{\gamma +af(H_0,0,0)}\right) \bigg ]\textrm{d}x \\\le & {} \int _{\varOmega }\bigg [-\lambda \delta e^{m\tau } r_2 \frac{(H-H_0)^2}{HH_0} -\frac{r_1 \delta }{k}r_2e^{m\tau }\left( (H-H_0) + I \right) ^2 \\{} & {} \quad + e^{m\tau } r_1 \delta \alpha I (\mathcal {R}_0(\tau ) -1)+D\frac{r_1 \delta f(H_0,0,0)}{\gamma +af(H_0,0,0)}\varDelta V\bigg ]\textrm{d}x. \end{aligned}$$

From the Divergence theorem and the homogeneous Neumann boundary conditions, we get

$$\begin{aligned} \int _{\varOmega }^{}\varDelta V\textrm{d}x=\int _{\partial \varOmega }^{}\frac{\partial V}{\partial n} =0, \end{aligned}$$

and thus, the previous inequality becomes

$$\begin{aligned} \frac{d L_{1}(t)}{\textrm{d}t}\le & {} \int _{\varOmega }^{} \bigg (-\lambda \delta e^{m\tau } r_2 \frac{(H-H_0)^2}{HH_0} -\frac{r_1 \delta }{k}r_2e^{m\tau }\left( (H-H_0) + I \right) ^2 \\{} & {} \quad + e^{m\tau } r_1 \delta \alpha I \Big (\mathcal {R}_0(\tau ) -1\Big )\bigg )\textrm{d}x. \end{aligned}$$

Therefore, the condition $$\mathcal {R}_0(\tau ) <1$$ ensures $$\frac{d L_{1}(x,t)}{\textrm{d}t} \le 0 $$, for all *H*, *I*, *V* $$ \ge 0$$. Furthermore, $$\frac{d L_{1}(t)}{\textrm{d}t} = 0 $$ if and only, if $$H = H_{0}$$, $$I = 0$$, $$ V= 0$$. It then follows that the largest compact invariant set *G* of $$\left\{ (H,I,V) \in \mathbb {R}^{3}_{+} \,/\; \; \frac{d L_{1}}{\textrm{d}t}(t)=0\right\} $$ is $$\{E_{0}\}$$. By LaSalle’s invariance principle [7], Theorem  5.3.1], we deduce that the spatially homogeneous uninfected equilibrium $$E_{0}$$ of the Reaction-Diffusion-ODE model (2) is globally asymptotically stable. This completes the proof. $$\square $$

#### Remark 3.3

The previous results confirm that the infection always dies out. These results strictly extend those of [4, 11, 32] in the case where their models ignored cell proliferation and the absorption effect in the asymptotic stability result of the homogeneous uninfected equilibrium.

### Numerical results

In this section, we present the numerical simulations done by using Matlab software to confirm the theoretical results that we established in the previous section for a particular case of the incidence function *f* defined as follows:

$$\begin{aligned} f(H,I,V)=\frac{\beta H}{1+\alpha _1 H+\alpha _2 V}, \end{aligned}$$

which is the Beddington–DeAngelis incidence function [13] where $\alpha _1$, $\alpha _2$ and $\beta$ are positive constants.

**Fig. 1:**
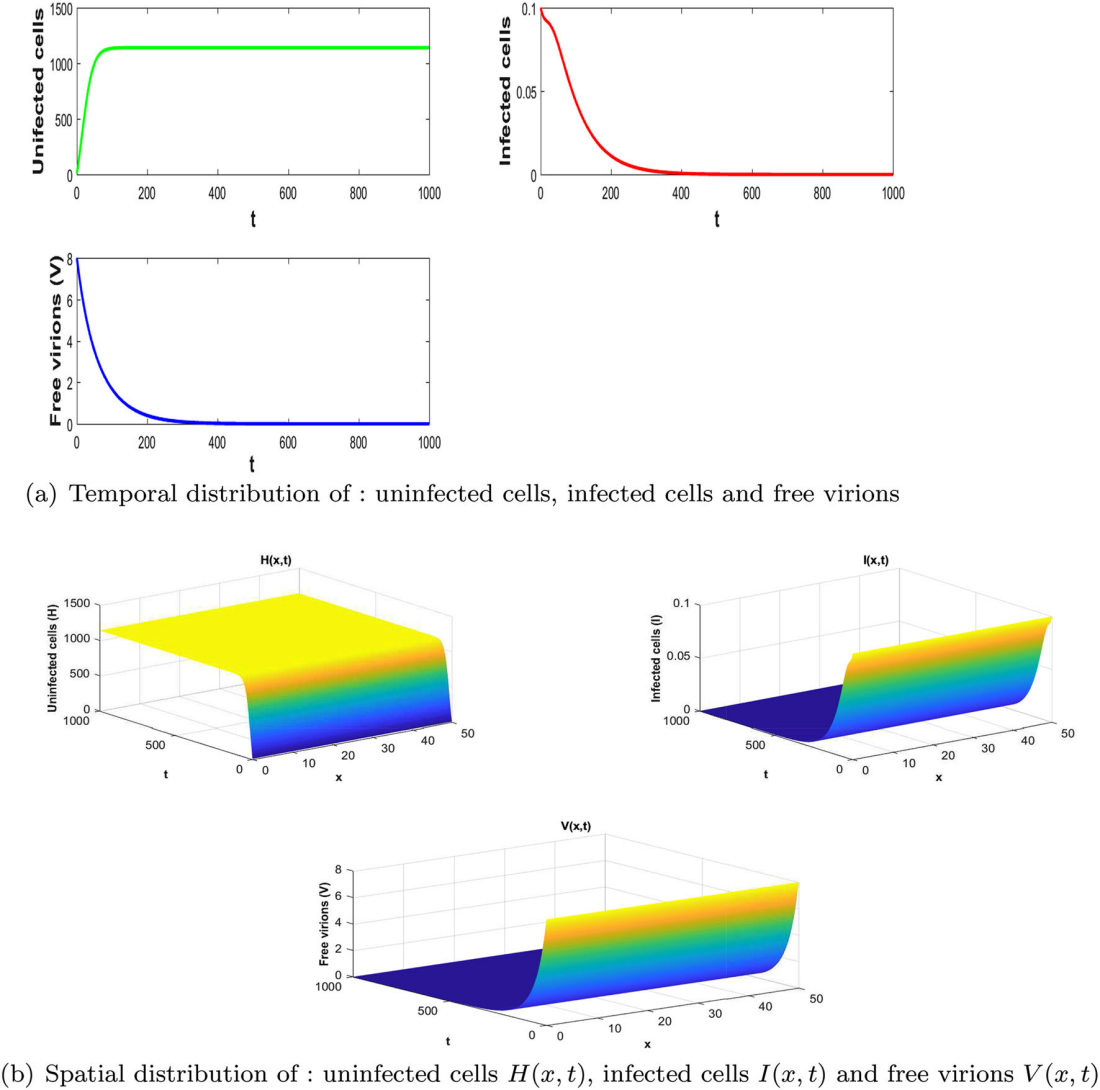
Demonstration of stability of the uninfected equilibrium with parameters: $$\tau = 5$$; $$r_1=0.05$$; $$ r_2=0.021$$; $$ m=0,021$$; $$k = 1200$$; $$a = 0.01$$; $$\beta =9.2419.10^{-7}$$; $$\mu =0.02$$; $$\gamma = 0.02$$; $$\alpha = 0.031$$; $$\lambda = 20 $$; $$\eta = 0.21$$; $$\alpha _1=0.001$$; $$\alpha _2=0.001$$; $$D=0.01$$. Here $$H_0=1140.8$$, $$\mathcal {R}_0=0.1836<1$$ and $$E_{0}= (1140, 0, 0)$$

## Asymptotic stability analysis of the infected equilibrium

The aim of this section is: first to determine the existence and the uniqueness of the infected equilibrium for the system (2), then to study the local and global stability of the the later, and finally to obtain numerical results. Due to the complexity of determining an infected equilibrium of the initial model, we will in this section restrict ourselves to the case $a = 0$.

### Infected equilibrium

#### Existence

##### Proposition 4.1

Assume that $\mathcal {R}_0 (\tau ) >1$, then the system (2) possesses an infected equilibrium $E_1=(H_1,I_1,V_1)$, where every component is strictly positive.

##### Proof

We suppose that there exists a homogeneous spatial equilibrium or constant solution $(H_{1}, I_{1}, V_{1})$ for system (2), then this constant solution satisfies: 
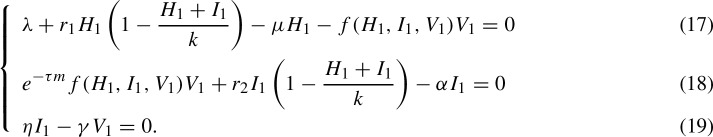
 From (19) we get

20 $$\begin{aligned} V_1=\frac{\eta }{\gamma } I_1. \quad \quad \end{aligned}$$

Moreover, by multiplying (17) by $e^{-m\tau }$ and adding the latter to (18), we obtain the following quadratic equation in $H_{1}$:

21 $$\begin{aligned}{} & {} -\dfrac{r_1}{k}e^{-m\tau }H_1^2+\bigg (\left( r_1-\mu -\dfrac{r_1}{k}I_{1}\right) e^{-m\tau }-\frac{r_2}{k}I_1\bigg )H_1+\lambda e^{-m\tau }\nonumber \\{} & {} +r_{2}I_{1}\bigg (1-\frac{I_{1}}{k}\bigg )-\alpha I_1=0. \end{aligned}$$

Note that equation (21) has two real roots of opposite sign that depend on $I_{1}$. The positive real root of (21) is given by

$$\begin{aligned} H_1=\frac{\bigg (\left( r_1-\mu -\dfrac{r_1}{k}I_{1}\right) e^{-m\tau }-\frac{r_2}{k}I_1\bigg )+\sqrt{\varDelta }}{2\dfrac{r_1}{k}}, \end{aligned}$$

where

22 $$\begin{aligned} \varDelta =\bigg (\left( r_1-\mu -\dfrac{r_1}{k}I_{1}\right) e^{-m\tau }-\frac{r_2}{k}I_1\bigg )^{2}+4\dfrac{r_1}{k}e^{-m\tau }\bigg (\lambda e^{-m\tau }+r_{2}I_{1}\bigg (1-\frac{I_{1}}{k}\bigg )-\alpha I_1\bigg ). \end{aligned}$$

Defining $$H_1=h(I_1) $$ with

23 $$\begin{aligned} h(I_1)=\frac{\bigg (\left( r_1-\mu -\dfrac{r_1}{k}I_{1}\right) e^{-m\tau }-\frac{r_2}{k}I_1\bigg )+\sqrt{\varDelta }}{2\dfrac{r_1}{k}}. \end{aligned}$$

Substituting (19) and (23) into (18) yields

$$\begin{aligned} e^{-m\tau }f\left( h(I_1),I_1,\frac{\eta }{\gamma }I_1\right) \frac{\eta }{\gamma }I_1+r_{2}I_1\bigg (1-\frac{h(I_1)+I_1}{k}\bigg )-\alpha I_1=0. \end{aligned}$$

It follows that

$$\begin{aligned} e^{-m\tau }f\left( h(I_1),I_1,\frac{\eta }{\gamma }I_1\right) \frac{\eta }{\gamma }+r_{2}\bigg (1-\frac{h(I_1)+I_1}{k}\bigg )-\alpha =0 \end{aligned}$$

since $$I_1>0$$. Let

24 $$\begin{aligned} F(I_1)=e^{-m\tau }f\left( h(I_1),I_1,\frac{\eta }{\gamma }I_1\right) \frac{\eta }{\gamma }+r_{2}\bigg (1-\frac{h(I_1)+I_1}{k}\bigg )-\alpha . \end{aligned}$$

*F* is a continuous real function defined on $[0,+\infty )$. Furthermore,

$$\begin{aligned} F(0)=e^{-m\tau }f\left( h(0),0,0\right) \frac{\eta }{\gamma }+r_{2}\bigg (1-\frac{h(0)}{k}\bigg )-\alpha , \end{aligned}$$

and

$$\begin{aligned} h(0)=\dfrac{\left( r_1-\mu \right) e^{-m\tau }+\sqrt{\left( r_1-\mu \right) ^2e^{-2m\tau }+4\lambda e^{-2m\tau } \dfrac{r_1}{k}}}{2\dfrac{r_1 e^{-m\tau }}{k}}=H_0. \end{aligned}$$

Finally, we get

$$\begin{aligned} F(0)=\alpha \bigg [\frac{e^{-m\tau }f(H_0,0,0)\eta }{\alpha \gamma }+\frac{r_{2}}{\alpha }\bigg (1-\frac{H_0}{k}\bigg )-1 \bigg ]. \end{aligned}$$

Therefore

25 $$\begin{aligned} F(0)= \alpha \left( \mathcal {R}_0(\tau ) - 1\right) \end{aligned}$$

which is positive as $\mathcal {R}_0 (\tau ) >1$. Note that

$$\begin{aligned} 0\le \lim \limits _{I_1 \rightarrow +\infty }h(I_1)<+\infty . \end{aligned}$$

Using the fact that there exists $\beta >0$ such that $f(H,I,V)\le \beta H$ for all *H*, *I*, *V* $\ge 0$, we deduce that

$$\begin{aligned} \lim \limits _{I_1 \rightarrow +\infty }f\left( h(I_1), I_1,\frac{\eta }{\gamma }I_1\right) <+\infty \end{aligned}$$

and therefore

$$\begin{aligned} \lim \limits _{I_1 \rightarrow +\infty }F(I_1)=-\infty . \end{aligned}$$

The intermediate value theorem guarantees the existence of $I_1>0$ such that $F(I_1)=0$. The existence of $I_1$ also ensures that of $H_1$ and of $V_1$. Thus the infected equilibrium point $E_1 = (H_1, I_1, V_1)$ exists. $\square$

Now let’s take a look at uniqueness.

#### Uniqueness

##### Proposition 4.2

If $A=H^{2}_{0}r_2-\lambda k \le 0$, then the infected equilibrium point $E_1=(H_1,I_1,V_1)$ is unique.

##### Proof

It is sufficient to show that $F(I_{1})$ is a strictly decreasing function. We have

$$\begin{aligned} F'(I_1)= & {} e^{-m\tau }h'(I_1)\frac{\partial f}{\partial H}\left( h(I_1),I_1,\frac{\eta }{\gamma }I_1\right) \frac{\eta }{\gamma }+e^{-m\tau }\frac{\partial f}{\partial I}\left( h(I_1),I_1,\frac{\eta }{\gamma }I_1\right) \frac{\eta }{\gamma }\\{} & {} +e^{-m\tau }\frac{\partial f}{\partial V}\left( h(I_1),I_1,\frac{\eta }{\gamma }I_1\right) \left( \frac{\eta }{\gamma }\right) ^{2}-\frac{r_2}{k}-\frac{r_2}{k}h'(I_1) \\ {}= & {} e^{-m\tau }h'(I_1)\frac{\partial f}{\partial H}\left( h(I_1),I_1,\frac{\eta }{\gamma }I_1\right) \frac{\eta }{\gamma }+e^{-m\tau }\frac{\partial f}{\partial I}\left( h(I_1),I_1,\frac{\eta }{\gamma }I_1\right) \frac{\eta }{\gamma }\\ {}{} & {} +e^{-m\tau }\frac{\partial f}{\partial V}\left( h(I_1),I_1,\frac{\eta }{\gamma }I_1\right) \left( \frac{\eta }{\gamma }\right) ^{2}-\frac{r_2}{k}(h'(I_1)+1). \end{aligned}$$

Note that $F'(I_{1})$ depends on $h'(I_{1})$. To calculate $h'(I_{1})$ we first rewrite the Eq. (21) as

26 $$\begin{aligned}{} & {} -\dfrac{r_1}{k}e^{-m\tau }h(I_1)+\bigg (\left( r_1-\mu -\dfrac{r_1}{k}I_{1}\right) e^{-m\tau }-\frac{r_2}{k}I_1\bigg ) \nonumber \\{} & {} +\frac{1}{h(I_1)}\bigg (\lambda e^{-m\tau }+r_{2}I_{1}\bigg (1-\frac{I_{1}}{k}\bigg )-\alpha I_1\bigg )=0. \end{aligned}$$

Using implicit differentiation we get

$$\begin{aligned} h'(I_1)= & {} -\left[ \frac{r_1}{k}e^{-m\tau }+\frac{r_2}{k}+\frac{1}{h(I_1)}\left( \alpha -r_2\bigg (1-\frac{2I_1}{k}\bigg )\right) \right] \\{} & {} \times \left[ \frac{r_1}{k}e^{-m\tau }+\frac{1}{h^{2}(I_1)}\bigg (\lambda e^{-m\tau }+r_{2}I_{1}\bigg (1-\frac{I_{1}}{k}\bigg )-\alpha I_1\bigg )\right] ^{-1}. \end{aligned}$$

The previous expression of $h'(I_{1})$ can be rewritten as

$$\begin{aligned} h'(I_1)=\frac{A}{B}, \end{aligned}$$

where

$$\begin{aligned} A=-\frac{r_1}{k}e^{m\tau }h^{2}(I_1)+r_2h(I_1)\bigg (1-\frac{h(I_1)+I_1}{k}\bigg )-\alpha h(I_1)-\frac{r_2}{k}I_1h(I_1) \end{aligned}$$

and

$$\begin{aligned} B=\frac{r_1}{k}e^{m\tau }h^{2}(I_1)+\lambda e^{-m\tau }+r_{2}I_{1}\bigg (1-\frac{I_{1}}{k}\bigg )-\alpha I_1 +\frac{r_2}{k}I_1h(I_1). \end{aligned}$$

Using Eqs. (18) and (19) we rewrite A and B respectively as

$$\begin{aligned} A=-\frac{r_1}{k}e^{m\tau }h^{2}(I_1)-h(I_1)e^{-m\tau }f(h(I_1),I_1,\frac{\eta }{\gamma }I_1) \frac{\eta }{\gamma }-\frac{r_2}{k}I_1 h(I_1) \end{aligned}$$

and

$$\begin{aligned} B=\frac{r_1}{k}e^{m\tau }h^{2}(I_1)+\lambda e^{-m\tau }-e^{-m\tau }f\left( h(I_1),I_1,\frac{\eta }{\gamma }I_1\right) \frac{\eta }{\gamma }I_1+\frac{r_2}{k}I_1 h(I_1). \end{aligned}$$

Note that

27 $$\begin{aligned} A+B=\lambda e^{-m\tau }-e^{-m\tau }f\left( h(I_1),I_1,\frac{\eta }{\gamma }I_1\right) \frac{\eta }{\gamma }I_1-h(I_1)e^{-m\tau }f\left( h(I_1),I_1,\frac{\eta }{\gamma }I_1\right) \frac{\eta }{\gamma }. \end{aligned}$$

From (27), we deduce that

$$\begin{aligned} F'(I_1)= & {} e^{-m\tau }\frac{A}{B}\frac{\partial f}{\partial H}\left( h(I_1),I_1,\frac{\eta }{\gamma }\right) \frac{\eta }{\gamma } +e^{-m\tau }\frac{\partial f}{\partial I}\left( h(I_1),I_1,\frac{\eta }{\gamma }\right) \frac{\eta }{\gamma }\\{} & {} +e^{-m\tau }\frac{\partial f}{\partial V}\left( h(I_1),I_1,\frac{\eta }{\gamma }\right) \left( \frac{\eta }{\gamma }\right) ^{2}\\{} & {} -\frac{r_2}{k B}\bigg (\lambda -f\left( h(I_1),I_1,\frac{\eta }{\gamma }\right) \frac{\eta }{\gamma }I_1\bigg )e^{-m\tau }+ \frac{r_2}{k B} h(I_1)f\left( h(I_1),I_1,\frac{\eta }{\gamma }\right) \frac{\eta }{\gamma }\bigg )e^{-m\tau }\\\le & {} e^{-m\tau }\frac{A}{B}\frac{\partial f}{\partial H}\left( h(I_1),I_1,\frac{\eta }{\gamma }\right) \frac{\eta }{\gamma } -\frac{r_2}{k B}\bigg (\lambda -f\left( h(I_1),I_1,\frac{\eta }{\gamma }\right) \frac{\eta }{\gamma }I_1\bigg )e^{-m\tau }\\ {}{} & {} + \frac{r_2}{k B} h(I_1)f\left( h(I_1),I_1,\frac{\eta }{\gamma }\right) \frac{\eta }{\gamma }\bigg )e^{-m\tau }. \end{aligned}$$

From (17), we have

$$\begin{aligned} \bigg (\lambda -f\left( h(I_1),I_1,\frac{\eta }{\gamma }\right) \frac{\eta }{\gamma }I_1 \bigg )e^{-m\tau }\ge \bigg (h^{2}(I_1)\frac{\lambda }{H^{2}_0}+ \frac{r_1}{k}I_1h(I_1)\bigg )e^{-m\tau }, \end{aligned}$$

from which we deduce that

$$\begin{aligned} F'(I_1)\le & {} e^{-m\tau }\frac{A}{B}\frac{\partial f}{\partial H}\left( h(I_1),I_1,\frac{\eta }{\gamma }\right) \frac{\eta }{\gamma } -\frac{r_2}{k B}\bigg (h^{2}(I_1)\frac{\lambda }{H^{2}_0}+ \frac{r_1}{k}I_1h(I_1)\bigg )e^{-m\tau }\\ {}{} & {} + \frac{r_2}{k B} h(I_1)f\left( h(I_1),I_1,\frac{\eta }{\gamma }\right) \frac{\eta }{\gamma }\bigg )e^{-m\tau }\\\le & {} \frac{r_2}{k B} \frac{h(I_1)}{I_1}\bigg (f\left( h(I_1),I_1,\frac{\eta }{\gamma }\right) \frac{\eta }{\gamma }I_1- \frac{\lambda }{H^{2}_0}h(I_1)I_1- \frac{r_1}{k}I^{2}_1\bigg )e^{-m\tau }\\ {}{} & {} +e^{-m\tau }\frac{A}{B}\frac{\partial f}{\partial H}\left( h(I_1),I_1,\frac{\eta }{\gamma }\right) \frac{\eta }{\gamma }. \end{aligned}$$

Equation (18), allows us to have:

$$\begin{aligned} F'(I_1)\le & {} \frac{r_2}{k B} \frac{h(I_1)}{I_1}\bigg ((\alpha -r_2)I_1+\frac{(r_2-r_1)}{k}I^{2}_1+\frac{r_2}{k}h(I_1)I_1- \frac{\lambda }{H^{2}_0}h(I_1)I_1\bigg )e^{-m\tau }\\ {}{} & {} +e^{-m\tau }\frac{A}{B}\frac{\partial f}{\partial H}\left( h(I_1),I_1,\frac{\eta }{\gamma }\right) \frac{\eta }{\gamma }\\\le & {} \frac{r_2}{k B} \frac{h(I_1)}{I_1}\bigg (\alpha -r_2\bigg (1-\frac{(H^{2}_0 r_2-\lambda k) h(I_1)}{k H^{2}_0 r_2}\bigg )\bigg )I_1e^{-m\tau } +e^{-m\tau }\frac{A}{B}\frac{\partial f}{\partial H}\left( h(I_1),I_1,\frac{\eta }{\gamma }\right) \frac{\eta }{\gamma }\\< & {} 0. \end{aligned}$$

Therefore

$$\begin{aligned} F'(I_1)<0. \end{aligned}$$

The fact that *F* is a strictly monotonic function allows us to conclude that the point $$I_1$$ is unique. The uniqueness of $$I_1$$ results in that of $$ H_1$$ and $$V_1$$. Thus we conclude that the equilibrium point $$ (H_1, I_1, V_1) $$ is unique. $$\square $$

##### Remark 4.3

Indeed, any spatially-inhomogeneous equilibrium point $E_{1}=(H_{1}, I_{1}, V_{1})$ of the model (2) subject to homogeneous Neumann boundary condition (4) must solve the following system 
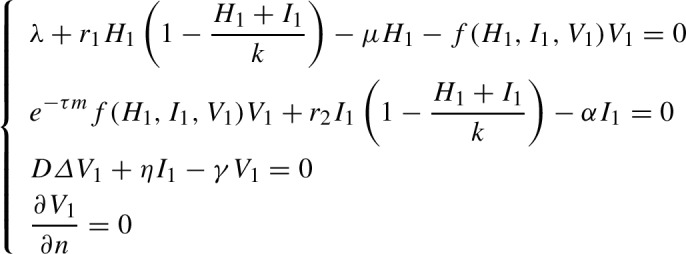
 Investigation of the existence and stability of such spatially-inhomogeneous equilibria will be the concern of a forthcoming work via an in-depth analysis of the above system.

### Local stability analysis of $E_{1}$

In this section we deal with the local stability of the infected equilibrium $E_{1}$.

#### Proposition 4.4

Let

$$\begin{aligned} B=\bigg (H_1\frac{\partial f(H_1,I_1,V_1)}{\partial H}-f(H_1,I_1,V_1)\bigg ). \end{aligned}$$

If $\mathcal {R}_0(\tau )>1$ and $B\ge 0$, then the infected equilibrium $E_{1}=(H_1,I_1,V_1)$, if it exists, is locally asymptotically stable.

#### Proof

Note that this proof is analogous to the proof of Proposition 3.1.

The characteristic equation of system (2) at the infected equilibrium is of the form

28 $$\begin{aligned} X^3 +a_2(\tau )X^2 + a_1(\tau )X+\big [b_2 (\tau )X^{2} + b_1(\tau )X+b_0(\tau )\big ] e^{-X\tau } + a_0(\tau )=0, \end{aligned}$$

where

$$\begin{aligned} a_2(\tau )= & {} \bigg (\frac{\lambda }{H_1}+\frac{r_1}{k}H_1 +\frac{\partial f(H_1,I_1,V_1)}{\partial H}V_1-f(H_1,I_1,V_1)\frac{V_1}{H_1}+ \gamma + \mu _{i}D \\{} & {} e^{-m\tau }f(H_1,I_1,V_1)\frac{V_1}{I_1} + \frac{r_2}{k}I_1 \bigg ), \\ a_{1}(\tau )= & {} \Bigg [\bigg (\gamma + \mu _{i}D +e^{-m\tau }f(H_1,I_1,V_1)\frac{V_1}{I_1} + \frac{r_I}{k}I_1 \bigg )\bigg (\frac{\lambda }{H_1}+\frac{r_1}{k}H_1 +\frac{\partial f(H_1,I_1,V_1)}{\partial H}V_1 \\{} & {} \quad -f(H_1,I_1,V_1)\frac{V_1}{H_1}\bigg )+\bigg ( +e^{-m\tau }f(H_1,I_1,V_1)\frac{V_1}{I_1} + \frac{r_I}{k}I_1 \bigg )\bigg (\gamma + \mu _{i}D\bigg )\\{} & {} \quad -\bigg (\frac{r_1}{k}H_1+\frac{\partial f(H_1,I_1,V_1)}{\partial V}V_1\bigg )\frac{r_2}{k}I_1 \bigg ], \\ a_0(\tau )= & {} \bigg (\frac{\lambda }{H_1} + \frac{r_1}{k} H_1+\frac{\partial f(H_1,I_1,V_1)}{\partial H}V_1-f(H_1,I_1,V_1)\frac{V_1}{H_1} \bigg ) \bigg [\bigg ( e^{-m\tau }f(H_1,I_1,V_1)\frac{V_1}{I_1} + \frac{r_2}{k}I_1 \bigg )\times \\{} & {} \quad \bigg (\gamma + \mu _{i}D\bigg )\bigg ]- \bigg (\frac{r_1}{k}H_1+\frac{\partial f(H_1,I_1,V_1)}{\partial I}V_1\bigg )\frac{r_2}{k} I_1(\gamma +\mu _{i}D)\\{} & {} \quad -\bigg (f(H_1,I_1,V_1)+\frac{\partial f(H_1,I_1,V_1)}{\partial V}V_1\bigg )\frac{r_2}{k}\eta I_1, \\ b_{2}(\tau )= & {} -e^{-m\tau }\frac{\partial f(H_1,I_1,V_1)}{\partial I}V_1, \\ b_1(\tau )= & {} -\bigg [\eta e^{-m\tau }f(H_1,I_1,V_1)+ \eta e^{-m\tau }\frac{\partial f(H_1,I_1,V_1)}{\partial V}V_1-\bigg (\frac{r_1}{k} H_1+\frac{\partial f(H_1,I_1,V_1)}{\partial I}V_1\bigg )\times \\{} & {} \quad e^{-m\tau }\frac{\partial f(H_1,I_1,V_1)}{\partial H}V_1 +\bigg (e^{-m\tau }\frac{\partial f(H_1,I_1,V_1)}{\partial I}V_1\bigg )\bigg [\bigg (\gamma +\mu _{i}D\bigg )+\\{} & {} \quad \bigg (\frac{\lambda }{H_1}+\frac{r_1}{k}H_1 +\frac{\partial f(H_1,I_1,V_1)}{\partial H}V_1-f(H_1,I_1,V_1)\frac{V_1}{H_1}\bigg )\bigg ]\bigg ], \end{aligned}$$

and

$$\begin{aligned} b_0(\tau )= & {} -\bigg (\frac{\lambda }{H_1} + \frac{r_1}{k} H_1+\frac{\partial f(H_1,I_1,V_1)}{\partial H}V_1-f(H_1,I_1,V_1)\frac{V_1}{H_1} \bigg )\bigg [\bigg (\eta e^{-m\tau }f(H_1,I_1,V_1)\\{} & {} \quad +\eta e^{-m\tau }\frac{\partial f(H_1,I_1,V_1)}{\partial V}V_1+\bigg (e^{-m\tau }\frac{\partial f(H_1,I_1,V_1)}{\partial I}V_1\bigg )\bigg (\gamma +\mu _{i}D\bigg )\bigg ]+\bigg (\frac{r_1}{k} H_1+\\{} & {} \quad \frac{\partial f(H_1,I_1,V_1)}{\partial I}V_1 \bigg )\bigg (\gamma +\mu _{i}D\bigg ) e^{-m\tau }\frac{\partial f(H_,I_1,V_1)}{\partial H}V_1 +\bigg (f(H_1,I_1,V_1)+\frac{\partial f(H_1,I_1,V_1)}{\partial V}V_1\bigg )\times \\{} & {} \quad \bigg (\eta e^{-m\tau }\frac{\partial f(H_1,I_1,V_1)}{\partial H}V_1\bigg ). \end{aligned}$$

When $\tau = 0$, Eq. (28) becomes

29 $$\begin{aligned} X^3 +(a_2(0)+ b_2 (0))X^2 + (a_1(0)+ b_1(0))X+b_0(0) + a_0(0)=0. \end{aligned}$$

By the Routh-Hurwitz criterion the conditions for real part of X $\mathcal {R}_e X$ to be negative are: $a_2(0)+ b_2(0) > 0$, $b_0(0) + a_0(0)> 0$, and $(a_2(0)+b_2(0))(a_1(0) + b_1(0)) - (a_0(0) + b_0(0) ) > 0$.

In our case we have

$$\begin{aligned} a_2(0)+b_2(0)= & {} \frac{\lambda }{H_1}+\frac{r_1}{k}H_1 +\frac{\partial f(H_1,I_1,V_1)}{\partial H}V_1-f(H_1,I_1,V_1)\frac{V_1}{H_1}+ \gamma + \mu _{i}D \\ {}{} & {} + \frac{r_2}{k}I_1 +f(H_1,I_1,V_1)\frac{V_1}{I_1} -\frac{\partial f(H_1,I_1,V_1)}{\partial I}V_1>0, \end{aligned}$$

and

$$\begin{aligned} a_0(0)+b_0(0)= & {} \bigg (\frac{\lambda }{H_1} -f(H_1,I_1,V_1)\frac{V_1}{H_1} \bigg ) \bigg [\frac{r_2}{k}I_1 \bigg (\gamma + \mu _{i}D\bigg )\bigg ] +\frac{\partial f(H_1,I_1,V_1)}{\partial H}V_1\frac{r_2}{k}I_1\mu _{i}D\\{} & {} \quad +\bigg (\frac{\lambda }{H_1} + \frac{r_1}{k} H_1+\frac{\partial f(H_1,I_1,V_1)}{\partial H}V_1-f(H_1,I_1,V_1)\frac{V_1}{H_1} \bigg ) f(H_1,I_1,V_1)\frac{V_1}{I_1} \mu _{i}D\\{} & {} \quad - \frac{\partial f(H_1,I_1,V_1)}{\partial I}V_1\frac{r_2}{k} I_1(\gamma +\mu _{i}D) -\frac{\partial f(H_1,I_1,V_1)}{\partial V}V_1\frac{r_2}{k}\eta I_1 \\ {}{} & {} \quad -\bigg (\frac{\lambda }{H_1} + \frac{r_1}{k} H_1-f(H_1,I_1,V_1)\frac{V_1}{H_1} \bigg ) \eta \frac{\partial f(H_1,I_1,V_1)}{\partial V}V_1 \\{} & {} \quad -\bigg (\frac{\lambda }{H_1} + \frac{r_1}{k} H_1-f(H_1,I_1,V_1)\frac{V_1}{H_1} \bigg ) \bigg (\frac{\partial f(H_1,I_1,V_1)}{\partial I}V_1\bigg )\bigg (\gamma +\mu _{i}D\bigg ) \\ {}{} & {} \quad +\frac{r_1}{k} H_1\mu _{i}D \frac{\partial f(H_,I_1,V_1)}{\partial H}V_1 +\eta f(H_1,I_1,V_1) \frac{\partial f(H_1,I_1,V_1)}{\partial H}V_1\\{} & {} \quad \frac{\gamma V_1}{k}\bigg (r_2\frac{\partial f(H_1,I_1,V_1)}{\partial H}I_1+r_1\frac{\partial f(H_1,I_1,V_1)}{\partial H}H_1-r_2f(H_1,I_1,V_1)\bigg ) >0, \end{aligned}$$

since $B > 0$, so we need

$$\begin{aligned} H(1):\quad a_0(0)+b_0(0)> 0, \quad (a_2(0)+b_2(0))(a_1(0) + b_1(0)) - (a_0(0) +b_0(0) ) > 0. \end{aligned}$$

If $\tau = 0$, by the Routh-Hurwitz criterion, we have the following result. $\square$

#### Proposition 4.5

If conditions H(1) are satisfied, $B=\bigg (\frac{\partial f(H_1,I_1,V_1)}{\partial H}H_1-f(H_1,I_1,V_1)\bigg ) \ge 0$, and $\mathcal {R}_0(0)>1$, then the infected equilibrium $E_{1}=(H_1,I_1,V_1)$ is locally asymptotically stable.

Now we analyze if it is possible to have a complex root with positive real part for the case $\tau > 0$, assuming *H*(1) satisfies, note that $X = 0$ is not a root of (28) because $a_0(\tau ) + b_0(\tau ) > 0$. Now suppose that $X= i \omega$, with $\omega > 0$, is a root of (28) so the next equation must be satisfied by $\omega$

$$\begin{aligned}{} & {} -i\omega ^3 - a_2(\tau ) \omega ^2 + ia_1(\tau )\omega + a_0(\tau ) -b_2(\tau )\omega ^{2}\cos \omega \tau +ib_2(\tau )\omega ^{2}\sin \omega \tau + i b_1(\tau )\omega \cos \omega \tau \\{} & {} +b_1(\tau )\omega \sin \omega \tau +b_0(\tau )\omega \cos \omega \tau - i b_0(\tau )\sin \omega \tau = 0. \end{aligned}$$

Separating the real and the imaginary parts of the previous expression yields: 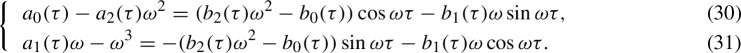 From (30) and (31), we get

$$\begin{aligned} \cos \omega \tau =\frac{ (-a_2(\tau )b_2(\tau )+b_1(\tau ))\omega ^{4}+(a_0(\tau )b_2(\tau )+a_2(\tau )b_0(\tau )-a_1(\tau )b_1(\tau ))\omega ^{2}-a_0(\tau )b_0(\tau ) }{(b_2(\tau )\omega ^{2}- b_0(\tau ))^{2}+b_1(\tau )\omega ^{2}} \end{aligned}$$

and

$$\begin{aligned} \sin \omega \tau =\frac{b_2(\tau ) \omega ^{5}+(-b_0(\tau )-a_1(\tau )b_2(\tau )+a_2(\tau )b_1(\tau ))\omega ^{3}+ (a_1(\tau )b_0(\tau )-a_0(\tau )b_1(\tau ))\omega }{(b_2(\tau )\omega ^{2}- b_0(\tau ))^{2}+b_1(\tau )\omega ^{2}}. \end{aligned}$$

Moreover, note that the characteristic Eq. (28) is equivalent to

32 $$\begin{aligned} P(X,\tau ) + Q(X,\tau )e^{-X\tau } = 0 \end{aligned}$$

with

$$\begin{aligned} P(X,\tau )= & {} X^3 + a_2(\tau )X^2 + a_1(\tau ) + a_0(\tau ),\\ Q(X,\tau )= & {} b_2(\tau ) X^{2}+b_1(\tau ) X + b_0(\tau ). \end{aligned}$$

It follows from the above that:

$$\begin{aligned} \sin (\omega \tau ) = \mathcal {I}_m \frac{Q(i\omega , \tau )}{P(i\omega , \tau )} \end{aligned}$$

and

$$\begin{aligned} \cos (\omega \tau ) = -\mathcal {R}_e \frac{Q(i\omega , \tau )}{P(i\omega , \tau )}. \end{aligned}$$

Hence, we conclude that $\omega$ is a positive root of the following equation:

$$\begin{aligned} |P(i\omega , \tau )|^2=|Q(i\omega , \tau )|^2 \end{aligned}$$

that is:

33 $$\begin{aligned} \omega ^6 -A \omega ^4 + B\omega ^2 + C=0 \end{aligned}$$

where

$$\begin{aligned} A= & {} a^{2}_2(\tau )-b^{2}_2(\tau ) -2a_1(\tau ),\\ B= & {} a_1^2(\tau )- 2a_0(\tau )a_2(\tau ) - b_1^2(\tau )+2b_2(\tau )b_{0}(\tau ),\\ C= & {} a_0^2(\tau ) -b_{0}^{2}(\tau )). \end{aligned}$$

Let $z=\omega ^2$ then (33) becomes the third order equation in *z*

34 $$\begin{aligned} z^{3}+Az^{2}+Bz+C=0. \end{aligned}$$

Suppose that (34) has at least one positive root, let $z_{0}$ be the smallest value for these roots. Then (33) has the root $\omega _0 = \sqrt{z_0}$ then, according to (30) and (31), we obtain the value of $\tau$ associated with this $\omega _0$ such that $X = \omega i$ is an purely imaginary root of (32), known as

$$\begin{aligned} \tau _0= \frac{1}{\omega _0} \arccos \left[ \frac{ (a_2\omega ^{2}_0-a_0 ) +b_2\omega ^{2}_0(a_0 -a_2\omega ^{2}_0) + b_1 \omega _0 (\omega _0^3 - a_1 \omega _0)}{(b_2\omega ^{2}_0- b_0 )^{2}+b_1\omega ^{2}_0} \right] . \end{aligned}$$

Then we have the following result, from Lemma  2.1 from Ruan [24].:

#### Theorem 4.6

Assume (*H*(1)),

1. If $C\ge 0$ and $\varLambda = A^2 - 3B<0$, then all roots of (32) have negative real part for all $\tau \ge 0$ and the infected equilibrium $E_1=(H_1, I_1, V_1)$ is locally asymptotically stable.
2. If $C<0$ or $C\ge 0$, $z_1>0$ and $z_1^3 + Az_1^2 + Bz_1 + C\le 0$, then all roots of (32) have negative real parts when $\tau \in [0, \tau _0]$ and the infected equilibrium $E_1=(H_1,I_1,V_1)$ is locally asymptotically stable in $[0,\tau _0]$.

### Global stability analysis of $E_{1}$

Next, we study the global stability of the infected equilibrium without absorption of $E_1 =(H_1,I_1,V_1)$ for the model problem (2)–(4). We assume that

$$\begin{aligned}{} & {} (\mathcal {H}_{5}): \left( 1 - \frac{f(H,I,V)}{ f(H,I_1,V_1)}\right) \left( \frac{f(H,I_1,V_1)}{ f(H,I,V)}-\frac{V}{V_1}\right) \le 0,\,\,\, H,I,V\ge 0,\\{} & {} \quad (\mathcal {H}_{6}): \left( 1 - \frac{f(H_1,I_1,V_1)}{f(H,I_1,V_1)}\right) =\frac{\delta _2(H-H_1)}{H},\,\,\, H,\delta _2>0. \end{aligned}$$

Biological interpretations of $(\mathcal {H}_{5})$ can be easily found in [15].

#### Theorem 4.7

If $r_1=r_2=r$ $e^{m\tau }-3\le 0$, $-\left( \delta _2-\frac{1}{4}\left( 1+\frac{\delta _2}{e^{m \tau }}\right) ^{2}\right) \le 0$ and $-\bigg (\frac{1}{P H_1}(\lambda \delta _2 -f(H_1,I_1,V_1)V_1)-\frac{2r\delta _2}{k}\bigg )<0$ then the uninfected equilibrium $E_1 =(H_1,I_1,V_1)$, if it exists, is globally asymptotically stable.

#### Proof

Let *H*(*x*, *t*), *I*(*x*, *t*) and *V*(*x*, *t*) be any positive solution of the delayed Reaction–Diffusion-O.D.E model problem (2)–(4). Define the following Lyapunov functional

35 $$\begin{aligned} L_2(t) =\int _{\varOmega }^{}\left( \tilde{L}(x,t) + f(H_1,I_1,V_1)V_1 L_{+}(x,t)\right) \textrm{d}x, \end{aligned}$$

where

$$\begin{aligned} \tilde{L}(x,t)= & {} \left( H-H_1- \int _{H_1}^{H} \frac{f(H_1,I_1,V_1)}{f(\eta ,H_1,V_1)} \textrm{d}\eta \right) + e^{m\tau }\int _{I_1}^{I} \frac{\eta - I_1}{\eta } \textrm{d}\eta \\{} & {} + \frac{f(H_1,I_1,V_1)V_1}{\eta I_1} \int _{V_1}^{V} \frac{\eta -V_1}{\eta }\textrm{d}\eta \end{aligned}$$

and

$$\begin{aligned} L_{+}(x,t)= & {} \int _{0}^{\tau } \Bigg ( \frac{f(H(x,t-\omega ),I(x,t-\omega ), V(x,t-\omega ))V(x,t-\omega ))}{f(H_1,I_1,V_1)V_1} - 1 \\{} & {} - \ln \frac{f(H(x,t-\omega ),I(x,t-\omega ) V(x,t-\omega ))V(x,t-\omega ) }{f(H_1, I_1, V_1)V_1}\Bigg )d\omega . \end{aligned}$$

The time derivative of $L_+$ is given by:

$$\begin{aligned} \frac{\partial L_{+}(x,t)}{\partial t}= & {} - \frac{ f(H_{\tau },I_{\tau }, V_{\tau })V_{\tau }}{f(H_1,I_1,V_1)V_1} +\frac{f(H,I, V)V }{f(H_1,I_1, V_1)V_1} + \ln \frac{f(H_{\tau },I_{\tau }, V_{\tau })V_{\tau }}{f(H_1,I_1, V_1)V_1}\\{} & {} - \ln \frac{f(H,I, V)V}{f(H_1,I_1, V_1)V_1}\\= & {} - \frac{ f(H_{\tau },I_{\tau }, V_{\tau })V_{\tau }}{f(H_1,I_1,V_1)V_1} +\frac{f(H,I, V)V }{f(H_1,I_1, V_1)V_1 } + \ln \frac{H_1}{H} +\ln \frac{f(H_{\tau },I_{\tau }, V_{\tau })V_{\tau }I_1}{f(H_1,I_1, V_1)V_1 I}\\{} & {} + \ln \frac{f(H_1,I_1,V_1)V_1H I }{f(H,I, V)V H_1 I_1}. \end{aligned}$$

Noting that

$$\begin{aligned} \ln \frac{f(H_1,I_1,V_1)V_1H I }{f(H,I, V)V H_1 I_1}= & {} \ln \frac{f(H_1,I_1,V_1)f(H,I_1,V_1)V_1H I }{f(H,I, V)f(H,I_1,V_1)V H_1 I_1}\\ {}= & {} \ln \frac{f(H,I_1,V_1)}{f(H,I, V)}+\ln \frac{V_1I}{VI_1}+ \ln \frac{f(H_1,I_1,V_1)H}{ f(H,I_1,V_1)H_1}. \end{aligned}$$

Inserting the previous equation in the expression of $\frac{\partial L_{+}(x,t)}{\partial t}$, one deduces that

$$\begin{aligned} \frac{\partial L_{+}(x,t)}{\partial t}= & {} - \frac{f(H_{\tau },I_{\tau }, V_{\tau })V_{\tau }}{f(H_1,I_1,V_1)V_1} +\frac{f(H,I, V)V }{f(H_1,I_1, V_1)V_1 } + \ln \frac{H_1}{H}+ \ln \frac{f(H_{\tau },I_{\tau }, V_{\tau })V_{\tau }I_1}{f(H_1,I_1, V_1)V_1 I}\\{} & {} \quad +\ln \frac{f(H,I_1,V_1)}{f(H,I, V)}+ \ln \frac{f(H_1,I_1,V_1)H}{ f(H,I_1,V_1)H_1}+\ln \frac{V_1I}{VI_1}. \end{aligned}$$

Likewise,

$$\begin{aligned} \frac{\partial \tilde{L}(x,t)}{\partial t}= & {} \left( 1-\frac{f(H_1,I_1,V_1)}{f(H,I_1,V_1)}\right) \left( \lambda -\frac{r}{k}H(H + I) -f(H,I,V)V + (r - \mu )H \right) \\{} & {} \quad +e^{m\tau } (I-I_1) \left( e^{-m\tau }\frac{f(H_{\tau },I_{\tau }, V_{\tau })V_{\tau }}{I} - \frac{r}{k}(H+I) + r - \alpha \right) \\{} & {} \quad + \frac{ f(H_1,I_1,V_1)V_1}{\eta I_1 }\left( 1 - \frac{V_1}{V}\right) \left( \eta I -\gamma V +D\varDelta V\right) . \end{aligned}$$

Since $(H_1, I_1, V_1)$ is an infected equilibrium, we get:

$$\begin{aligned} r - \mu= & {} - \frac{\lambda }{H_1} + \frac{r}{k}(H_1 + I_1)+\frac{f(H_1,I_1,V_1)V_1}{H_1}, \\ r - \alpha= & {} \frac{r}{k}(H_1 + I_1) - \frac{e^{-m\tau } f(H_1,I_1,V_1)V_1 }{I_1},\\ \gamma= & {} \frac{\eta I_1}{V_1}. \end{aligned}$$

Using the previous inequalities, we have:

$$\begin{aligned} \frac{\partial \tilde{L}(x,t)}{\partial t}= & {} \delta _2 (H-H_1)\Bigg ( \frac{\lambda }{H} - \frac{r}{k}(H+I) -\frac{\lambda }{H_1} + \frac{r}{k}(H_1 + I_1)\Bigg )+\left( 1-\frac{f(H_1,I_1,V_1)}{f(H,I_1,V_1)}\right) \times \\{} & {} \quad \left( -f(H,I,V)V+ \frac{f(H_1,I_1,V_1)V_1 H}{ H_1}\right) + e^{m\tau } (I- I_1) \Bigg (\frac{e^{-m\tau } f(H_{\tau },I_{\tau },V_{\tau })V_{\tau }}{I} \\{} & {} \quad -\frac{r}{k}(H+I) + \frac{r}{k}(H_1 + I_1) - \frac{e^{-m\tau } f(H_1,I_1,V_1) V_1}{I_1 }\Bigg ) + \frac{ f(H_1,I_1,V_1)V_1}{\eta I_1 }\times \\{} & {} \quad \left( 1 - \frac{V_1}{V}\right) \left( \frac{\eta (I V_1 - I_1 V)}{V_1} \right) + \frac{ f(H_1,I_1,V_1)V_1}{\eta I_1}\left( 1 - \frac{V_1}{V}\right) D\varDelta V, \end{aligned}$$

which can be written as:

$$\begin{aligned} \frac{\partial \tilde{L}(x,t)}{\partial t}= & {} \delta _2 (H-H_1) \Bigg (\frac{-\lambda (H-H_1)}{H H_1} - \frac{r}{k}[(H-H_1)+(I-I_1)] \Bigg )\\{} & {} \quad +\left( 1-\frac{f(H_1,I_1,V_1)}{f(H,I_1,V_1)}\right) \left( -f(H,I,V)V+ \frac{f(H_1,I_1,V_1)V_1 H}{ H_1}\right) + (I-I_1)\times \\{} & {} \quad \Bigg (\frac{ f(H_{\tau },I_{\tau },V_{\tau })V_{\tau }}{I}- \frac{f(H_1,I_1,V_1) V_1}{I_1} - \frac{r}{k}e^{m\tau }\left( (H-H_1)+ (I-I_1)\right) \Bigg )\\{} & {} \quad + \frac{ f(H_1,I_1,V_1)V_1}{\eta I_1 }\left( 1 - \frac{V_1}{V}\right) \left( \frac{\eta I V_1 - I_1 V}{V_1} \right) + \frac{ f(H_1,I_1,V_1)V_1}{\eta I_1}\left( 1 - \frac{V_1}{V}\right) D\varDelta V. \end{aligned}$$

Hence

$$\begin{aligned} \frac{\partial \tilde{L}(x,t)}{\partial t}= & {} -\lambda \delta _2 \frac{(H-H_1)^2}{H H_1} + \frac{r}{k}e^{m\tau }\Big ( - (I - I_1)^2 - (H-H_1) (I - I_1) \\{} & {} \quad - \frac{\delta _2}{e^{m \tau }} (H - H_1)(I - I_1)-\frac{1}{4}\left( 1+\frac{\delta _2}{e^{m\tau }}\right) ^{2} (H - H_1)^2 \Big )+\frac{2r }{k}\delta _2(H-H_1)^{2}\\{} & {} \quad -\frac{r e^{m\tau }}{k}\left( \delta _2-\frac{1}{4}\left( 1+\frac{\delta _2}{e^{m \tau }}\right) ^{2}\right) (H-H_1)^{2}+\frac{r }{k}\delta _2\left( -3+e^{m\tau }\right) (H-H_1)^{2} \\{} & {} \quad + f(H_1,I_1,V_1)V_1 \Bigg (- \frac{f(H,I,V)V}{f(H_1,I_1,V_1)V_1}+ \frac{ f(H_{\tau },I_{\tau },V_{\tau })V_{\tau }}{f(H_1,I_1,V_1)V_1}\Bigg )\\{} & {} \quad + f(H_1,I_1,V_1)V_1 \Bigg (2+\frac{H}{H_1} -\frac{ f(H_{\tau },I_{\tau },V_{\tau })V_{\tau }I_1}{If(H_1,I_1,V_1)V_1}+\frac{f(H,I,V)V}{f(H,I_1,V_1)V_1}\Bigg )\\{} & {} \quad + f(H_1,I_1,V_1)V_1\Bigg ( -\frac{V}{V_1} -\frac{f(H_1,I_1,V_1)H}{f(H,I_1,V_1)H_1}-\frac{V_1I}{VI_1} \Bigg )\\{} & {} \quad + \frac{ f(H_1,I_1,V_1)V_1}{\eta I_1}\left( 1 - \frac{V_1}{V} \right) D\varDelta V\\= & {} -\lambda \delta _2 \frac{(H-H_1)^2}{H H_1} - \frac{ r e^{m\tau }}{k} \bigg [(I - I_1)+\frac{1}{2}\left( 1+\frac{\delta _2}{e^{m \tau }}\right) (H - H_1)\bigg ]^{2}+\frac{2r }{k}\delta _2(H-H_1)^{2}\\{} & {} \quad -\frac{r e^{m\tau }}{k}\left( \delta _2-\frac{1}{4}\left( 1+\frac{\delta _2}{e^{m \tau }}\right) ^{2}\right) (H-H_1)^{2}+\frac{r }{k}\delta _2\left( -3+e^{m\tau }\right) (H-H_1)^{2}\\{} & {} \quad + f(H_1,I_1,V_1)V_1 \Bigg (- \frac{f(H,I,V)V}{f(H_1,I_1,V_1)V_1}+ \frac{ f(H_{\tau },I_{\tau },V_{\tau })V_{\tau }}{f(H_1,I_1,V_1)V_1}-\frac{f(H_1,I_1,V_1)H}{f(H,I_1,V_1)H_1}\Bigg ) \\{} & {} \quad + f(H_1,I_1,V_1)V_1\bigg (5-\frac{H_1}{H} -\frac{V_1I }{V I_1}-\frac{ f(H_{\tau },I_{\tau },V_{\tau })V_{\tau }I_1}{If(H_1,I_1,V_1)V_1}-\frac{f(H,I_1,V_1)}{f(H,I,V)}\bigg )\\{} & {} \quad +f(H_1,I_1,V_1)V_1\Bigg ( -1-\frac{V}{V_1} + \frac{f(H,I_1,V_1)}{f(H,I,V)} + \frac{f(H,I,V)V}{f(H,I_1,V_1)V_1}\Bigg ) \\{} & {} \quad + f(H_1,I_1,V_1)V_1\bigg (\frac{H_1}{H}+\frac{H}{H_1}-2\bigg ) + \frac{ f(H_1,I_1,V_1)V_1}{\eta I_1}\left( 1 - \frac{V_1}{V }\right) D\varDelta V. \end{aligned}$$

Moreover,

$$\begin{aligned} \frac{H_1}{H} + \frac{H}{H_1}-2 = \frac{(H-H_1)^2}{H H_1}. \end{aligned}$$

Thus

$$\begin{aligned} \frac{\partial \tilde{L}(x,t)}{\partial t}= & {} \bigg (-\lambda \delta _2 +f(H_1,I_1,V_1)V_1\bigg ) \frac{(H-H_1)^2}{H H_1} - \frac{ r e^{m\tau }}{k} \bigg [(I - I_1)+\frac{1}{2}\left( 1+\frac{\delta _2}{e^{m \tau }}\right) (H - H_1)\bigg ]^{2}\\{} & {} \quad +\frac{2r }{k}\delta _2(H-H_1)^{2}-\frac{r e^{m\tau }}{k}\left( \delta _2-\frac{1}{4}\left( 1+\frac{\delta _2}{e^{m \tau }}\right) ^{2}\right) (H-H_1)^{2}+\frac{r }{k}\delta _2\left( -3+e^{m\tau }\right) (H-H_1)^{2} \\{} & {} \quad +f(H_1,I_1,V_1)V_1 \Bigg (- \frac{f(H,I,V)V}{f(H_1,I_1,V_1)V_1}+ \frac{ f(H_{\tau },I_{\tau },V_{\tau })V_{\tau }}{f(H_1,I_1,V_1)V_1}-\frac{f(H_1,I_1,V_1)H}{f(H,I_1,V_1)H_1}\Bigg ) \\{} & {} \quad + f(H_1,I_1,V_1)V_1\bigg (5-\frac{H_1}{H} -\frac{V_1I }{V I_1}-\frac{ f(H_{\tau },I_{\tau },V_{\tau })V_{\tau }I_1}{If(H_1,I_1,V_1)V_1}-\frac{f(H,I_1,V_1)}{f(H,I,V)}\bigg )\\{} & {} \quad + f(H_1,I_1,V_1)V_1\left( 1 - \frac{f(H,I,V)}{f(H,I_1,V_1)}\right) \left( \frac{f(H,I_1,V_1)}{f(H,I,V)}-\frac{V}{V_1}\right) \\{} & {} \quad + \frac{ f(H_1,I_1,V_1)V_1}{\eta I_1}\left( 1 -\frac{V_1}{V}\right) D\varDelta V. \end{aligned}$$

Also, from the Divergence Theorem and the homogeneous Neumann boundary conditions and the results of work [10], we have

$$\begin{aligned} \int _{ \varOmega }^{}\frac{1}{V}\varDelta V=\int _{ \varOmega }^{}\frac{1}{V^{2}}\Vert \nabla V\Vert ^{2}\textrm{d}x. \end{aligned}$$

and

$$\begin{aligned} \int _{ \varOmega }\varDelta V = 0. \end{aligned}$$

Then combining the final expressions of $\frac{\partial \tilde{L}(x,t)}{\partial t}$ and $\frac{\partial L_{+}(x,t)}{\partial t}$, and furthermore, using assumption $-\bigg (\frac{1}{P H_1}(\lambda \delta _2 -f(H_1,I_1,V_1)V_1)-\frac{2r\delta _2}{k}\bigg )<0$, we get:

$$\begin{aligned}{} & {} \frac{d L_2(t)}{\textrm{d}t} = \int _{\varOmega }\left( \frac{\partial \tilde{L}(x,t) }{\partial t} + f(H_1,I_1,V_1)V_1 \frac{\partial \tilde{L}_{+}(x,t) }{\partial t}\right) \textrm{d}x,\\{} & {} \le \int _{\varOmega }^{}\Bigg [-\left( \frac{1}{P H_1}\left( \lambda \delta _2 -f(H_1,I_1,V_1)V_1\right) -\frac{2r\delta _2}{k}\right) (H-H_1)^2+\frac{r }{k}\delta _2\left( -3+e^{m\tau }\right) (H-H_1)^{2}\\{} & {} \quad - \frac{ r e^{m\tau }}{k} \bigg [(I - I_1)+\frac{1}{2}\left( 1+\frac{\delta _2}{e^{m \tau }}\right) (H - H_1)\bigg ]^{2} -\frac{r e^{m\tau }}{k}\left( \delta _2-\frac{1}{4}\left( 1+\frac{\delta _2}{e^{m \tau }}\right) ^{2}\right) (H-H_1)^{2} \\{} & {} \quad +f(H_1,I_1,V_1)V_1 \Bigg (- \frac{f(H,I,V)V}{f(H_1,I_1,V_1)V_1}+ \frac{ f(H_{\tau },I_{\tau },V_{\tau })V_{\tau }}{f(H_1,I_1,V_1)V_1}-\frac{f(H_1,I_1,V_1)H}{f(H,I_1,V_1)H_1}\Bigg )\\{} & {} \quad + f(H_1,I_1,V_1)V_1\bigg (5-\frac{H_1}{H} -\frac{V_1I }{V I_1}-\frac{ f(H_{\tau },I_{\tau },V_{\tau })V_{\tau }I_1}{If(H_1,I_1,V_1)V_1}-\frac{f(H,I_1,V_1)}{f(H,I,V)}\bigg )\\{} & {} \quad +\frac{ f(H_1,I_1,V_1)V_1}{\eta I_1}\left( 1 -\frac{V_1}{V}\right) D\varDelta V+f(H_1,I_1,V_1)\bigg ( \ln \frac{V_1I}{VI_1} \\{} & {} \quad - \frac{f(H_{\tau },I_{\tau } V_{\tau })V_{\tau }}{f(H_1,I_1,V_1)V_1} +\frac{f(H, I, V)V }{f(H_1,I_1, V_1)V_1 } + \ln \frac{H_1}{H}+ \ln \frac{f(H_{\tau },I_{\tau } V_{\tau })V_{\tau }I_1}{f(H_1,I_1, V_1)V_1 I}\\{} & {} \quad +\ln \frac{f(H,I_1,V_1)}{f(H,I, V)}+ \ln \frac{f(H_1,I_1,V_1)H}{ f(H,I_1,V_1)H_1}\bigg )\Bigg ]\textrm{d}x\\{} & {} \le \int _{\varOmega }^{}\Bigg [-\left( \frac{1}{P H_1}\left( \lambda \delta _2 -f(H_1,I_1,V_1)V_1\right) -\frac{2r\delta _2}{k}\right) (H-H_1)^2+\frac{r }{k}\delta _2\left( -3+e^{m\tau }\right) (H-H_1)^{2}\\{} & {} \quad - \frac{ r e^{m\tau }}{k} \bigg [(I - I_1)+\frac{1}{2}\left( 1+\frac{\delta _2}{e^{m \tau }}\right) (H - H_1)\bigg ]^{2} -\frac{r e^{m\tau }}{k}\left( \delta _2-\frac{1}{4}\left( 1+\frac{\delta _2}{e^{m \tau }}\right) ^{2}\right) (H-H_1)^{2} \\{} & {} \quad - f(H_1,I_1,V_1)V_1 \Bigg ( \frac{f(H,I_1,V_1)}{f(H,I,V)}-1-\ln \frac{f(H,I_1,V_1)}{f(H,I,V)}\Bigg ) \\{} & {} \quad - f(H_1,I_1,V_1)V_1 \Bigg (\frac{ f(H_1,I_1,V_1)H }{f(H,I_1,V_1)H_1 }-1-\ln \frac{ f(H_1,I_1,V_1)H }{f(H,I_1,V_1)H_1}\Bigg ) \\{} & {} \quad - f(H_1,I_1,V_1)V_1 \Bigg (\frac{ f(H_{\tau },I_{\tau },V_{\tau })V_{\tau }I_1}{If(H_1,I_1,V_1)V_1}-1-\ln \frac{ f(H_{\tau },I_{\tau },V_{\tau })V_{\tau }I_1}{If(H_1,I_1,V_1)V_1}\Bigg ) \\{} & {} \quad - f(H_1,I_1,V_1)V_1\bigg (\frac{H_1}{H} -1-\ln \frac{H_1}{H}\bigg ) - f(H_1,I_1,V_1)V_1\bigg (\frac{V_1I}{VI_1} -1-\ln \frac{V_1 I}{VI_1}\bigg )\Bigg ]\textrm{d}x\\{} & {} \quad +\int _{\varOmega }^{}\frac{ f(H_1,I_1,V_1)V_1}{\eta I_1}\left( 1 - \frac{V_1}{V }\right) D\varDelta V\textrm{d}x. \end{aligned}$$

The fact that the real function *g* defined by $g(x)= x-1 - \ln x$ is positive on $(0,+\infty )$, one deduces that:

$$\begin{aligned} \frac{dL_{2}(t)}{\textrm{d}t}\le 0. \end{aligned}$$

**Fig. 2:**
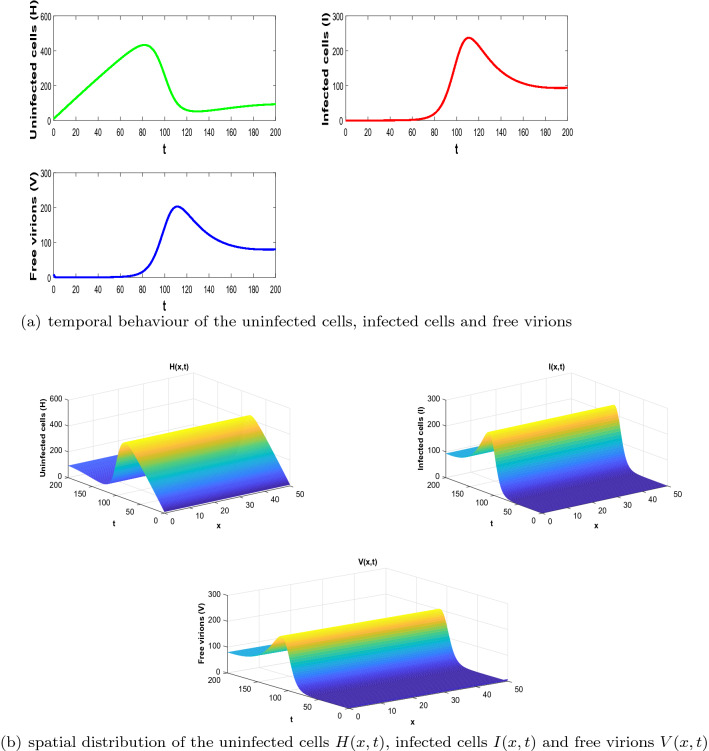
Demonstration of stability of the infected equilibrium with the set of parameter values: $$\tau = 10$$; $$r_1=0.001$$; $$ r_2=0.001$$; $$m=0.02$$; $$k = 1200$$; $$a = 0.01$$; $$\beta =0.00009$$; $$\mu =0.02$$; $$\gamma = 2.1$$; $$\alpha = 0.05$$; $$\lambda = 6 $$; $$\eta = 1.8$$; $$\alpha _1=0.001$$; $$\alpha _2=0.001$$; $$D=0.01$$. Here we find $$H_0=311.5328$$, $$\mathcal {R}_0=3.0153>1$$, $$E_1=(91.1385, 89.6691, 75.7164)$$

Therefore $$\frac{d L_{1}(t)}{\textrm{d}t} \le 0 $$, for all *H*, *I*, *V* $$ \ge 0$$. Furthermore, $$\frac{d L_{1}(t)}{\textrm{d}t} = 0 $$ if and only, if $$H = H_{1}$$, $$I = I_{1}$$, $$ V= V_{1}$$. It then follows that the largest compact invariant set *G* of $$\left\{ (H,I,V) \in \mathbb {R}^{3}_{+} \,/\; \; \frac{d L_{1}}{\textrm{d}t}=0\right\} $$ is $$\{E_{1}\}$$. By LaSalle’s invariance principle [7], Theorem  5.3.1, we deduce that the spatially homogeneous uninfected equilibrium $$E_{1}$$ of the PDE model system (2)–(4) is globally asymptotically stable. This completes the proof.

$$\square $$

#### Remark 4.8

Theorem 4.7 confirms that the infection persists. This result extends strictly those of [4, 11, 32] in that the cellular proliferation and absorption effect were ignored in establishing the result on the asymptotic stability of the homogeneous infected equilibrium.

### Numerical results

## Conclusion

In this work, we have proposed and analyzed a class of three dimensional spatio-temporal model describing infectious diseases caused by viruses such as the human immunodeficiency virus (HIV), hepatitis C virus and the hepatitis B virus (HBV). The infection transmission process is modeled by a general incidence function which includes several forms existing in the literature. In addition, the global analysis of the proposed model is rigorously investigated. Furthermore, biological findings of our analytical results are presented. Moreover, mathematical virus models and results presented in many previous studies are extended and generalized. To study the mechanism of viral infection and replication, we performed the mathematical analysis of a dynamic model of diffusive in-host virus with a general non-linear incidence function. The well-posedness and the stability of the equilibria of this model are examined. The basic reproductive number $\mathcal {R}_{0}(\tau )$ which is a threshold value that predicts extinction and persistence of the viral infection is given. It is shown that the global stability of the equilibria is determined by $\mathcal {R}_{0}(\tau )$ with some other conditions: if $\mathcal {R}_{0}(\tau ) < 1$, ​the uninfected equilibrium is globally asymptotically stable, which means that the virus is finally cleared and the infection dies; if $\mathcal {R}_{0}(\tau ) > 1$, then the infected equilibrium is globally asymptotically stable. Our results also imply that diffusion coefficients have no influence on the global behaviour of such a virus dynamics model with homogeneous Neumann boundary conditions. Furthermore, the model proposed in this work is an extension of some previous work and the results obtained improve some known results. It is interesting to improve the present work by integrating several delays and searching for the spatially heterogeneous equilibria. In addition, we can undertake the study of the existence of the Hopf bifurcation, and knowing that The memory is an important characteristic of biological systems, It will be more interesting to examine the memory effect on the spatiotemporal dynamics of our model by using the new generalized fractional derivative presented in [8].
